# Integrating genome and transcriptome profiling for elucidating the mechanism of muscle growth and lipid deposition in Pekin ducks

**DOI:** 10.1038/s41598-017-04178-7

**Published:** 2017-06-19

**Authors:** Liyuan Wang, Xiangxiang Li, Jun Ma, Yawen Zhang, Hao Zhang

**Affiliations:** 1 National Engineering Laboratory for Animal Breeding/Beijing Key Laboratory for Animal Genetic Improvement, China Agricultural University, Beijing, 100094 People’s Republic of China; 2 Beijing Zoo, Beijing, 100044 People’s Republic of China

## Abstract

Muscle growth and lipid deposition are co-ordinately regulated processes. Cherry Valley Pekin duck is a lean-type duck breed with high growth rate, whereas the native Pekin duck of China has high lipid deposition. Phenotypic analysis showed that native Pekin ducks have smaller fibre diameter and larger density in the breast muscle at 3 weeks of age and higher intramuscular fat content at 6 weeks of age than those in Cherry Valley Pekin ducks. We detected 17 positively selected genes (PSGs) by comparing genes mainly involved with muscle organ development, muscle contraction, peroxisome proliferator activated receptor signalling pathway, and fatty acid metabolism. In all, 52 and 206 differentially expressed genes (DEGs) were identified in transcriptomic comparisons between the two breeds at 3 and 6 weeks of age, respectively, which could potentially affect muscle growth and lipid deposition. Based on the integration of PSGs and DEGs and their functional annotations, we found that 11 and 10 genes were correlated with muscle growth and lipid deposition, respectively. Identification of candidate genes controlling quantitative traits of duck muscle might aid in elucidating the mechanisms of muscle growth and lipid deposition and could help in improving duck breeding.

## Introduction

Duck is an ancient domestic animal used for its meat, egg, and feather. Pekin duck is famous for its extensive adaptability, rapid growth during rearing stage, and superior taste rendered by the high content of intramuscular fat (IMF)^1^. The IMF content in the breast muscle of Pekin ducks that are fed ad libitum or are overfed is higher than that of similarly fed Muscovy ducks. Moreover, the breast muscle of Pekin ducks has lower lightness value, colour degree, and shearing force than those of Muscovy ducks, and the former is more tender, juicy, and less stringy^2^. The modern breeding system of duck primarily focuses on either the improvement of muscle growth or the preservation of abundant IMF content. Cherry Valley Pekin duck, a widely used meat duck breed, is a lean-type breed derived from Pekin duck, whereas the native Pekin duck in China has high lipid deposition ability. Therefore, these breeds are model systems for investigating the mechanisms underlying variation in muscle growth and lipid deposition under similar genetic backgrounds; the phenotypic differences between these two breeds might have resulted mostly from the differences in intensive artificial selection.

Both muscle growth and lipid deposition are complicated processes, which are accompanied by the proliferation, migration, and differentiation of somatic cells. Thus far, many candidate genes and gene families responsible for growth traits have been identified in duck; these include myogenic regulatory factor (MRF) family members [myogenic factor 5 (MYF5), muscle-specific regulatory factor 4 (MRF4), myoblast determination protein (MYOD), and myogenin (MYOG)^3^], myocyte enhancer factor-2 gene family (MEF2)^4^, lipoprotein lipase (LPL)^5^, and insulin-like growth factors (IGFs)^6^. Several pathways regulating lipid deposition, including proliferator-activated receptor-gamma (PPARγ), mitogen-activated protein kinase (MAPK), Wnt, Hedgehog, and CCAAT/enhancer bindings protein α (C/EBPα) signalling pathways, have also been reported^7, 8^.

A sustaining intensive selection on an animal population can generate phenotypic and the corresponding genetic changes. Phenotypic differences between two breeds with similar genetic backgrounds can be explained in terms of genomic variations and changes in their transcriptomes. Combined evaluation of the whole genome single nucleotide polymorphism (SNP) distribution by using whole-genome sequencing (WGS) and differentially expressed gene (DEG) profiles by using RNA-seq allows a better understanding of the genetic basis of different phenotypes and helps in the identification of candidate genes. Comprehensive studies involving WGS and RNA-seq have been performed for characters such as growth and lipid deposition in pigs^9, 10^ and egg quality in chicken^11^. However, to the best of our knowledge, no integrated WGS and transcriptome analysis has been reported in duck. An understanding of the genetic basis of different phenotypes in duck would be valuable in improving the efficiency and selection strategies involved in duck breeding.

In the present study, a comprehensive analysis combining WGS and RNA-seq was performed to determine the genomic variations and gene expression profiles for detecting candidate genes related to muscle growth and lipid deposition in Cherry Valley Pekin and native Pekin ducks.

## Results

### Measurement of IMF and muscle fibre in Cherry and Beijing ducks

**Figure 1:**
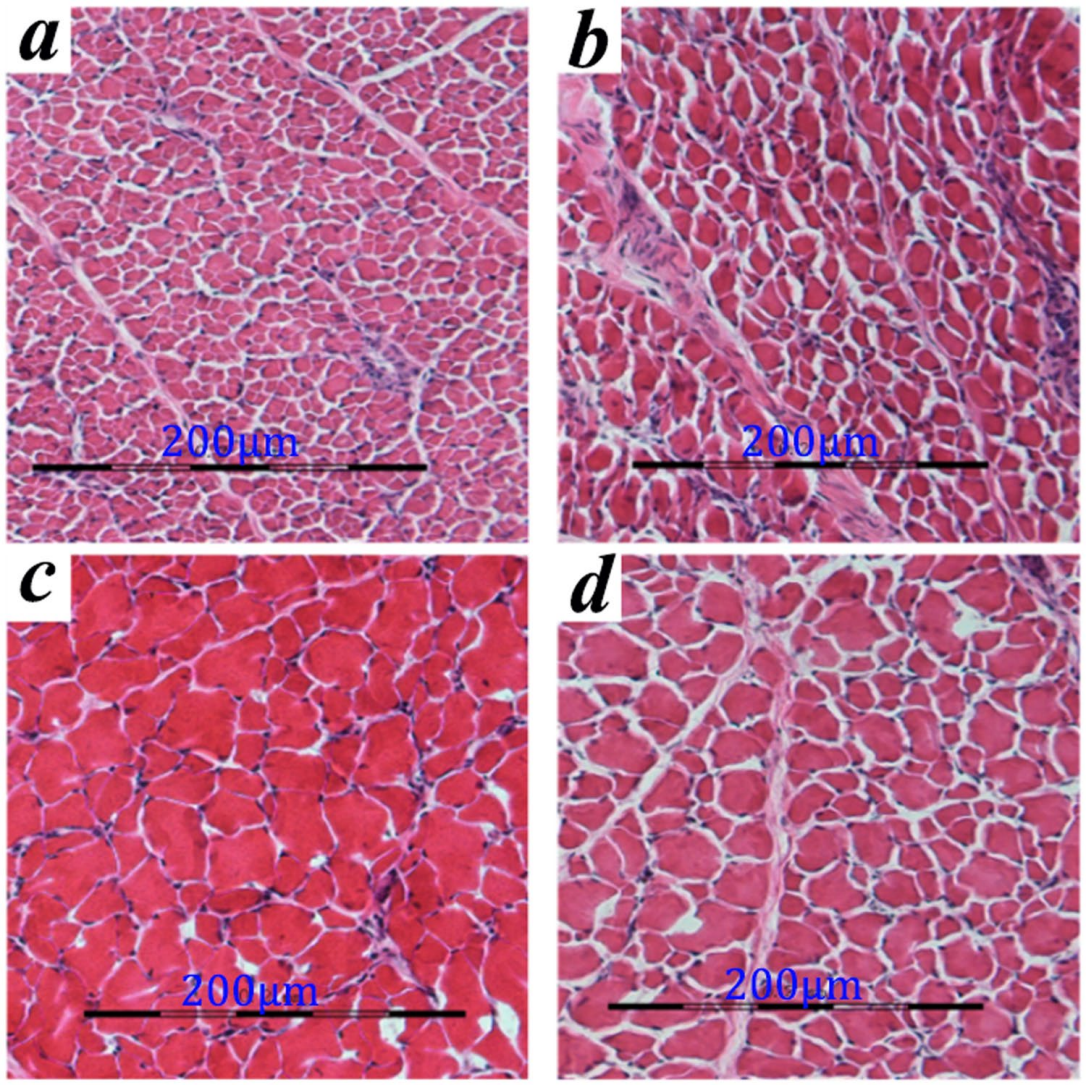
Histological analysis of breast muscles. (**a**) Cherry Valley Pekin ducks (CD) at 3 weeks of age, (**b**) Native Pekin ducks (BD) at 3 weeks of age, (**c**) CD at 6 weeks of age, (**d**) BD at 6 weeks of age; Scale bars: 200 μm.

Native Pekin ducks from Beijing (BD) had a higher IMF content than that in Cherry Valley Pekin ducks (CD) at 3 and 6 weeks of age, especially at 6 weeks (*p* < 0.05; Table 1). At 3 weeks, the diameter of muscle fibre was less and its density was more in BD compared to those in CD (*p* < 0.05; Table 1, Fig. 1). However, at 6 weeks, no significant differences were noted in these factors between the two breeds (*p* > 0.05).

**Table 1:** Phenotypic parameters measured in this study.

Population	Number of samples	Water content (%)	IMF (%)	Diameter of muscle fibre (μm)	Density of muscle fibre (1 × 10^3^/mm^2^)
BD 3 week	20	82.43 ± 0.20	0.1435 ± 0.008	5.85 ± 0.11	8.437 ± 0.317*
CD 3 week	20	83.67 ± 0.23	0.1332 ± 0.0102	11.91 ± 0.25*	3.106 ± 0.032
BD 6 week	20	76.98 ± 0.17	1.039 ± 0.12*	22.10 ± 0.65	1.342 ± 0.021
CD 6 week	20	77.70 ± 0.14*	0.5832 ± 0.049	22.94 ± 0.42	1.302 ± 0.019

Note: BD means native Pekin duck and CD means Cherry Valley Pekin duck. *Significant at *p* < 0.05. The two breeds were compared at the same time point.

### Estimation of genetic diversity between CD and BD

Whole genome sequencing of 18 ducks (9 BD and 9 CD) yielded 133.1 Gb clean data with an average of 7.394 Gb data per duck. The sequencing depth varied from 6.145 to 8.055, and Q30 distribution varied from 93.25 to 96.53 (see Supplementary Table S1). After genomic variations in the 18 individuals were screened, 4.2 million SNPs were identified. Among these, only about 159 K and 75 K SNPs were located in the promoter and exon regions, whereas most of them were located in the intron or intergenic regions (2.01 M and 1.96 M; see Supplementary Table S2).

**Figure 2:**
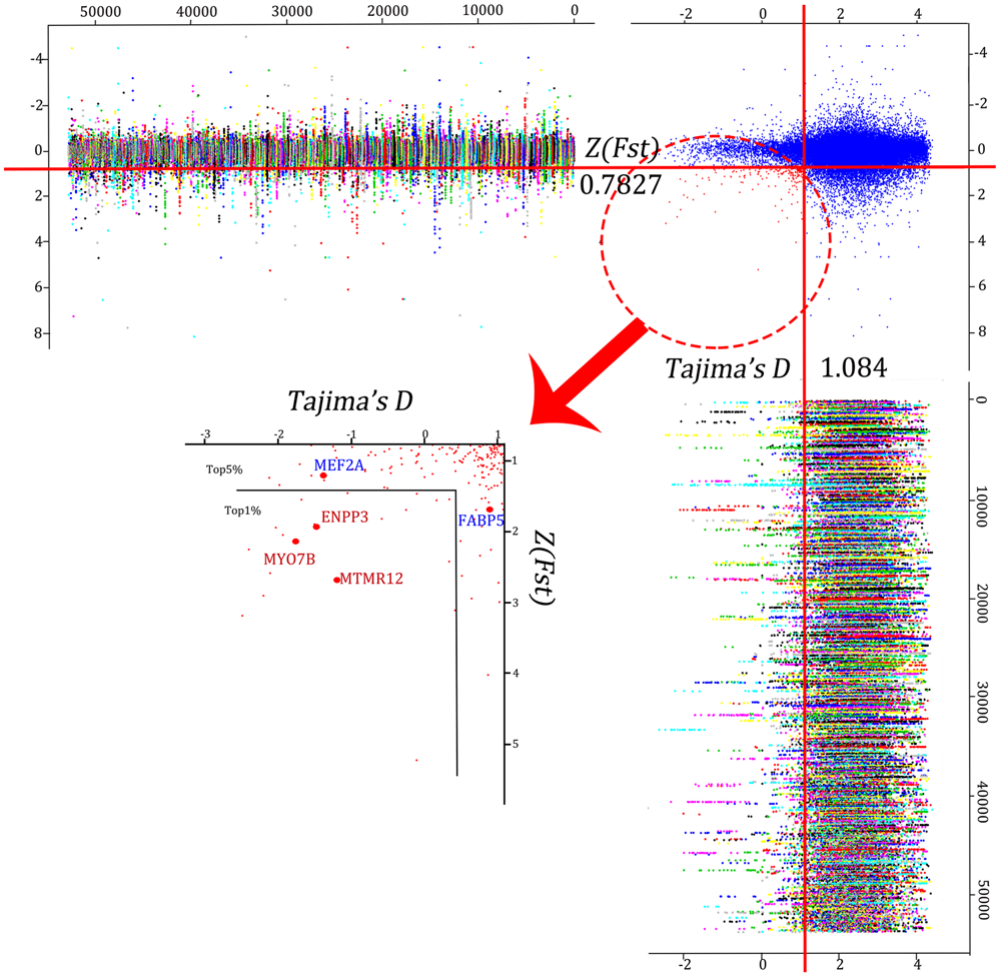
Distribution of positively selected genes calculated using a 40-K window along 1,592 scaffolds. Combination of Manhattan plotting of genomic differentiation Z(Fst) (upper left), Tajima’s D (lower right), and the overlap (upper right, lower left). MEF2A, myocyte enhancer factor 2A; FABP5, fatty acid binding protein 5; ENPP3, ectonucleotide pyrophosphatase/phosphodiesterase 3; MYO7B, myosin VIIB; MTMR12, myotubularin related protein 12.

**Figure 3:**
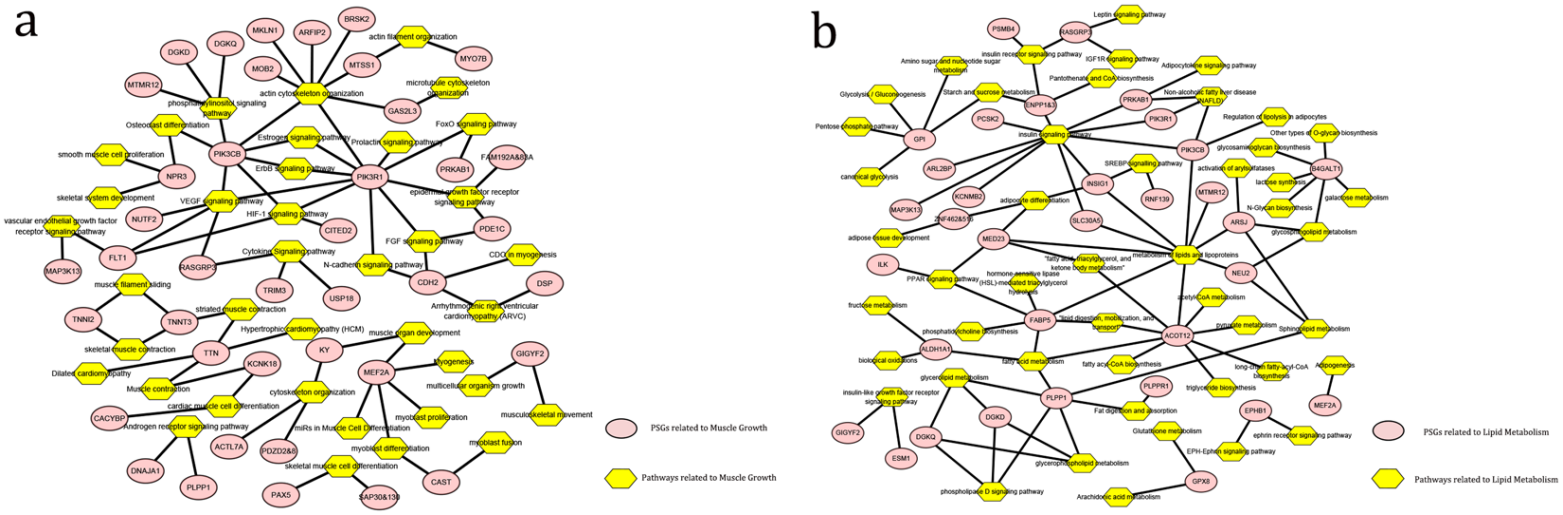
Visualization of main PSGs and pathways related to muscle growth and lipid metabolism. (**a**) PSGs and pathways related to muscle growth; (**b**) PSGs and pathways related to lipid metabolism. Note: ellipses stand for the main PSGs and rhombi stand for the main pathways.

Principal component analysis (PCA) revealed a clear distinction between BD and CD ducks (see Supplementary Figure S1), which was in accordance with the genetic background of the two breeds. Selection signatures were qualified in regions overlapped by two parameters with extremely high Z(Fst) values (top 5%, 0.7827; top 1%, 1.5718) and extremely low Tajima’s D values (top 5%, 1.084; top 1%, −0.5718) (Fig. 2). In all, 206 candidate genes from 224 positively selected windows were screened as positively selected genes (PSGs) that were widely spread along the scaffolds (see Supplementary Table S3). Based on functional cluster analysis, the 206 PSGs could be categorized into the following four groups: lipid metabolism-related, muscle growth-related, signal transduction-related, and immune-related (Fig. 3; see Supplementary Figure S2). Among these genes, myocyte enhancer factor 2 A (MEF2A), kyphoscoliosis peptidase (KY), and many others were functional genes that have been shown to participate in several main pathways related to muscle growth, such as those involved in muscle organ development and myoblast differentiation^12–14^. Insulin-induced gene 1 (INSIG1), ectonucleotide pyrophosphatase/phosphodiesterase (ENPP1 & 3), fatty acid-binding protein 5 (FABP5), and some other genes that regulate lipogenesis have also been reported previously^15–18^.

### Transcriptome analysis of the breast muscle in CD and BD

At least 4.6-Gb clean data, accounting for more than 90% of the raw data, were obtained in each group. Q20 and Q30, which are criterion for sequencing false-positive rates, ranged from 94.49% to 95.025% and 85.37% to 86.67%, respectively (see Supplementary Table S4). After the clean reads were mapped to the reference genome of *Anas platyrhynchos*, total mapped sequences were found to range from 64.03% to 69.94%, most (approximately 69–74.4%) of which were located within the exons (see Supplementary Table S5).

In all, 16,705 genes were detected to express in duck breast muscle tissues. Among these, 2,273 genes were regarded as novel genes; 14,496 genes could be annotated to the *A*. *platyrhynchos* database of BGI_duck_1.0. Further, 165, 96, 118, and 374 unique genes were solely detected in native Pekin ducks at 3 (BD3) and 6 weeks of age (BD6) and Cherry Valley Pekin ducks at 3 (CD3) and 6 weeks of age (CD6), respectively (see Supplementary Figure S3). Further, 14,562 genes were consistently detected in the four groups. Most of the genes were distributed between 0.1 to 1000 reads per kilobases per million reads (RPKM), and the four groups showed a similar read number distribution of genes (see Supplementary Figure S4).

**Figure 4:**
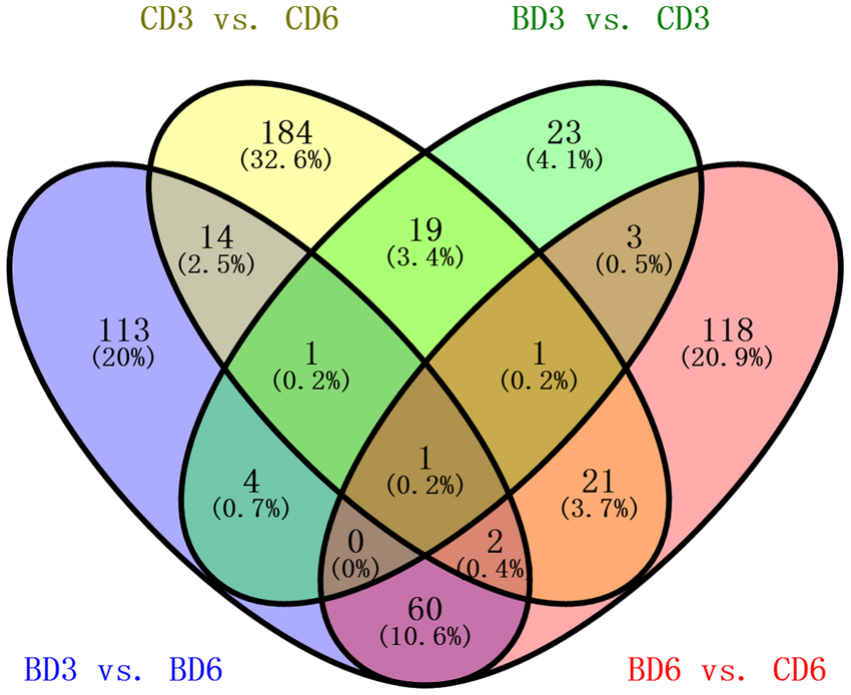
Distribution of DEGs between different time periods and breeds. BD3: 3-week-old native Pekin ducks; BD6: 6-week-old native Pekin ducks; CD6: 6-week-old Cherry Valley Pekin ducks; CD3: 3-week-old Cherry Valley Pekin ducks.

Total 564 DEGs were detected between the BD and CD and between 3- and 6-week-old (Fig. 4). Quantitative RT-PCR of 8 DEGs (ACSL1, CAV3, MYOG, TNNI1, MSTN, IGFBP5, FHL3, and CD36) validated that the DEGs were accurate and reliable (see Supplementary Figure S5). The expression profiles of DEGs identified by pairwise comparison of 3- and 6-week-old individuals of the two breeds were analysed. In all, 195 and 243 DEGs were identified in BD and CD, respectively, among which 126 and 121 genes were upregulated, and 69 and 122 genes were downregulated, respectively. Eighteen genes were common in the two breeds (Fig. 4). The analysis of biological functions indicated that the DEGs were involved in similar functions in the two breeds. Many of these genes were involved in muscle growth, for example, in muscle cell and muscle organ development, and a few were associated with lipid metabolism, such as in fatty acid metabolism and lipid localization. Other clustered genes were involved in processes related to energy metabolism, such as propanoate metabolism and GTPase activator activity (see Supplementary Tables S6, S7).

**Figure 5:**
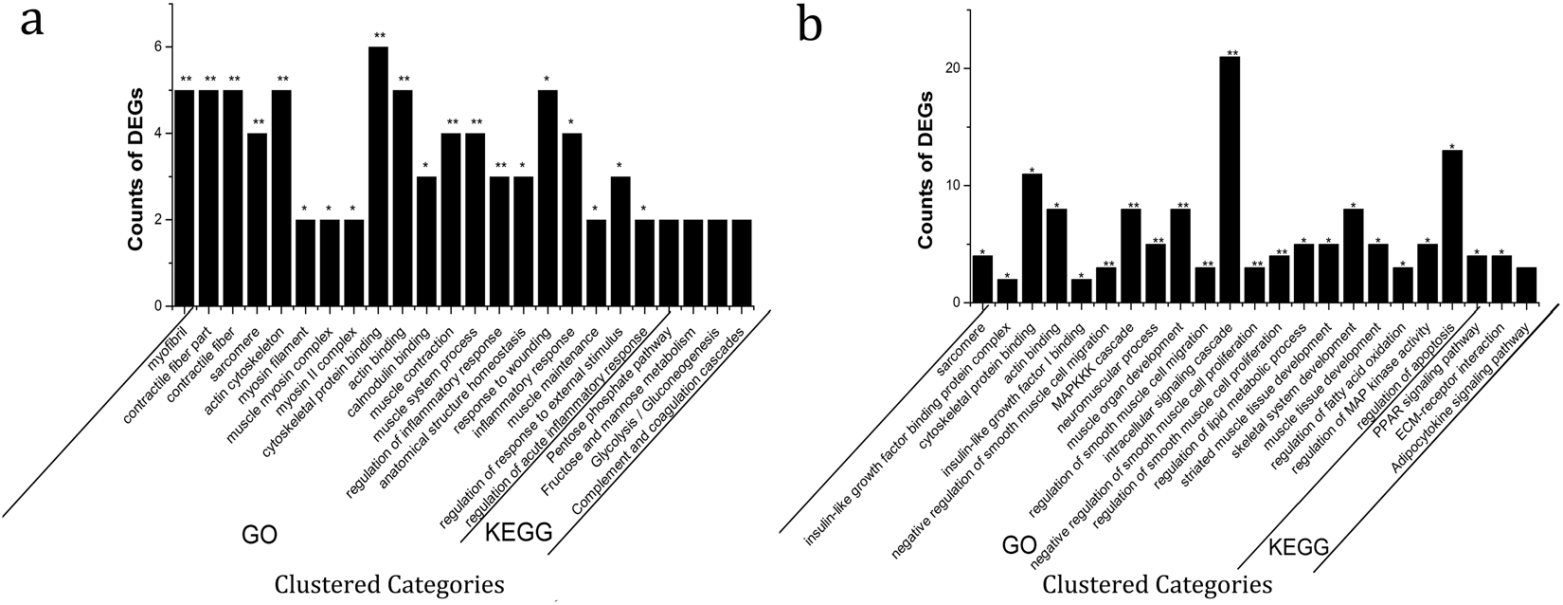
Biological analysis of DEGs identified between the two breeds. (**a**) BD3 vs. CD3, BD3: 3-week-old native Pekin ducks, CD3: 3-week-old Cherry Valley Pekin ducks; (**b**) BD6 vs. CD6, BD6: 6-week-old native Pekin ducks, CD6: 6-week-old Cherry Valley Pekin ducks. *Significant at *p* < 0.05; **significant at *p* < 0.01.

In all, 52 and 206 genes were differentially expressed between BD and CD at 3 and 6 weeks of age, respectively (Fig. 4). Between BD3 and CD3, most of the clusters were related to muscle growth, including cytoskeletal protein binding, muscle system process, and muscle maintenance, and the four most clustered pathways were all related to energy metabolism (Fig. 5a). Between BD6 and CD6, in addition to the muscle growth-related clusters, many clusters were also related to lipid metabolism, such as in the regulation of lipid metabolic process and fatty acid oxidation. PPAR signalling pathway, adipocytokine signalling pathway, and ECM-receptor interaction were also clustered in KEGG (p = 0.023, 0.039, and 0.051, respectively; Fig. 5b, Supplementary Table S7).

### Combination analysis of WGS and RNA-seq

Whole-genome sequencing was performed to detect genome variations underlying the phenotypic differences in muscle growth and lipid deposition between BD and CD. Genes differentially expressed between BD3 and CD3 were mainly regarded as muscle growth related (n = 55), because significant differences existed in the diameter and density of muscle fibres between BD and CD at 3 weeks of age. Moreover, DEGs between BD6 and CD6 could be largely distributed into lipogenesis-related genes (n = 206), since BD deposited considerably more lipid than CD at 6 weeks of age. DEGs between 3- and 6-week-old individuals were treated as breast growth-related genes involved in muscle and adipose development. After integrated analysis, 9 genes, which were both PSGs and DEGs, were confirmed as the candidate genes for breast growth (Table 2).

**Table 2:** Combination analysis of WGS and RNA-seq.

	DEG in BD3 vs. BD6	DEG in CD3 vs. CD6	DEG in BD3 vs. CD3	DEG in BD6 vs. CD6	Z(Fst)	Tajima’s *D*	Major pathways involved
PODXL	√	×	×	√	√	√	cell adhesion, integrin signalling pathway
ADGRG1	√	×	×	√	√	√	cell surface receptor signalling pathway, G-protein coupled receptor signalling pathway, angiogenesis
KY	×	×	√	×	√	√	muscle organ development, cytoskeleton organization
FABP5	√	×	×	√	√	√	PPAR signalling pathway, phosphatidylcholine biosynthesis, metabolism of lipids and lipoproteins, lipid digestion, mobilization, and transport, fatty acid metabolism,
FLT1	√	×	×	√	√	√	Cytokine-cytokine receptor interaction, HIF-1 signalling pathway, PI3K-Akt signalling pathway, Focal adhesion, vascular endothelial growth factor signalling pathway, MAPK signalling pathway
ENPP3	√	×	×	×	√	√	insulin signalling pathway, pantothenate and CoA biosynthesis, starch and sucrose metabolism,
OPN3	×	√	×	×	√	√	G-protein coupled receptor signalling pathway, circadian rhythm, GPCR signalling pathway
NGEF	√	×	×	×	√	√	cell death signalling pathway, EPH-Ephrin signalling pathway, GPCR signalling pathway, p75 NTR receptor-mediated signalling pathway
NEU2	√	×	×	√	√	√	sphingolipid metabolism, glycosphingolipid metabolism, metabolism of lipids and lipoproteins, sialic acid metabolism

√ Detected as DEG or PSG; × Not detected as DEG or PSG.

## Discussion

Muscle growth and lipid deposition have been reported to be regulated by a complex mechanism involving endogenous and exogenous factors. Several studies have investigated meat quality in many domesticated animals, including pig, cattle, and chicken^9–11^. Pekin duck is the common ancestor of current native Pekin and Cherry Valley Pekin ducks. Cherry Valley Pekin duck has been developed into a lean-type breed by modern breeding system, whereas native Pekin ducks in China have been allowed to preserve their high IMF content^5^. Phenotypic analysis showed that native Pekin ducks have lower breast muscle fibre diameter and higher fibre density than those of Cherry Valley Pekin ducks at 3 weeks of age; however, the IMF content was higher in the former at 6 weeks of age. Genomic variations result in phenotypic differences, and understanding the genetic basis of these phenotypes could contribute to the improvement of the breeding efficiency in duck. In this study, we conducted high quality whole-genome sequencing and transcriptome analysis of breast muscle at two key time points during the growth of native Pekin and Cherry Valley Pekin ducks in order to screen candidate genes regulating muscle growth and lipid deposition.

The loci under strong selective pressure are referred to as outliers and are always located at the extremes of empirical distribution. We used neutrality test (Tajima’s *D*) and fixation index Z(Fst) as the criteria for the detection of significant signatures as required. WGS detected a total of 206 candidate genes, among which 9 PSGs (ENPP3, FABP5, ADGRG1, NGEF, OPN3, FLT1, PODXL, KY, and NEU2) involved in pathways such as muscle organ development, muscle contraction, PPAR signalling, and fatty acid metabolism, were also differentially expressed in the transcriptome.

The transmembrane glycoprotein ENPP3 showed positive association with diabetes^19^. The higher content of ENPP3 reduced the serum ATP concentration as well as energy consumption, and thus facilitated lipid deposition^20^. Epidermal-type fatty acid-binding protein (E-FABP, FABP5), an intracellular protein that binds to lipid molecules and transports them to PPARs, was associated with fatty acid signalling, cell growth, and differentiation and was reported to play a protective role against oxidative damage to lipids^17, 18^. FABP5 was conservatively located close to fatty acid-binding protein 4 (FABP4, adipose) across the breeds, and SNP association analysis showed that FABP5 was strongly associated with fat deposition in a pig population^21^. Knockdown of FABP5 decreased the cellular cholesterol levels and led to the deposition of fewer lipid droplets in human and pigs^22, 23^. In addition, the expression level of FABP5 was correlated with those of other lipid regulators such as fatty acid desaturase 3 (FAD3) and metastasis markers in MCF cells^24^. Adhesion G protein-coupled receptor G1 (ADGRG1) belongs to the GPCR family, which mediates signal transduction across the cytomembrane. The three genes (ENPP3, FABP5, and ADGRG1) showed extremely high expression in BD6 ducks compared to those in BD3, CD3, and CD6, whereas no differences were observed in BD3 and CD3, indicating their key positive roles in IMF genesis. The association of neuronal guanine nucleotide exchange factor (NGEF) with ephrins receptors constitutes a molecular link between ephrins receptors and actin cytoskeleton, and a SNP in NGEF was found to be significantly associated with adipose deposition, both in the visceral adipose tissue and subcutaneous adipose tissue^25^. Opsin-3 (OPN3) has been shown to be involved in light sensation and circadian rhythms via the estrogen pathway^26^. OPN3 was also known to play a role in exocytosis of cytokine or steroid hormones. In the present study, NGEF and OPN3 decreased from 3 to 6 weeks of age, especially in BD ducks, indicating that they were likely the repressors of lipid genesis. Fms-related tyrosine kinase 1 (FLT1) is a class III receptor tyrosine kinase that plays important roles in hematopoiesis as a signal transduction protein^27^. FLT1 is also a direct receptor in the VEGF signalling pathway and thus has functions in the VEGF-related processes such as myogenic differentiation and endothelial targeting of lipids to peripheral tissues^28, 29^. The expression profiles of FLT1 evaluated in the present study showed a similar increase from 3 to 6 weeks of age in BD and CD; the increase was considerably higher in BD ducks, thereby confirming a complicated role of FLT1 in muscle growth and lipid deposition via VEGF. Podocalyxin (PODXL) is an adhesion stimulus protein that regulates cell adhesion and cytoskeletal organization along with insulin-like growth factor-binding proteins and integrin subunits^30^. PODXL-(+/+) populations of cells have a significantly faster growth rate than the PODXL-(−/−) populations^30^. KY, a cytoskeleton-associated protease, is essential for normal muscle growth, maturation, and stabilization. The KY protein was also reported to be linked to muscular dystrophy, and muscle fibre of KY-deficient mice was shown to grow abnormally^27^. Neuraminidase 2 (NEU2) enzymatic activity is causally linked to proper muscle differentiation, regeneration, and growth. NEU2-transfected myoblasts exhibited stronger muscle differentiation^31^. The expression of PODXL, KY, and NEU2 was higher in CD from age 3 to 6 weeks than that in BD. Taken together, these results suggest that PODXL, KY, and NEU2 were possibly the key regulators of muscle growth.

Whole-genome sequencing detected several other important genes (TTN, TNNI2, CDH2, MEF2A, PCSK1, INSIG1, and PLPPR1) with strong selection signal and differential expression tendency between the breeds in RNA-seq. Titin (TTN) interconnects the major structures of sarcomeres, including the M bands and Z discs and participates in a stress-sensing mechanism, thereby providing elasticity in conjunction with myomesin (MYOM), another PSG. TTN was also reported to be associated with muscle protein and contractile fibre content along with troponin I (TNNI2) and to play key roles in the structural organization of breast muscles. Certain SNPs, either in TNN or TNNIs, have been reported to be associated with meat and carcass trait^32^. N-cadherin (CDH2) plays an essential role in skeletal muscle differentiation through the N-cadherin-mediated adhesion process during the embryonic stages, since knockout animals showed heart defects and could be rescued by the re-expression of this protein specifically in the muscles^33^. A previous study on chicken identified the association between early morphogenetic events and N-cadherin level in the muscle, which might partly explain the phenotypic differences observed in the present study^34^. MEF2A, a member of the myocyte enhancer factor 2 transcription factor family, is essential for the specification and differentiation of the muscle lineage and is highly expressed in cardiac and skeletal muscles. MEF2A has been shown to facilitate skeletal muscle differentiation in humans^12^. Proprotein convertase subtilisin/kexin type 1 (PCSK1), which functions in the conversion of proinsulin to insulin as a signalling molecule, has been shown to have a significant correlation with pork meat quality, especially juiciness^35^. All the above-mentioned genes (TTN, TNNI2, CDH2, MEF2A, and PCSK1) were identified as muscle growth-related candidate genes in the present study by screening PSGs. Being an insulin-responsive gene, insulin-induced gene 1 protein (INSIG1) shows considerably more significant correlation with the marbling score and total fatty acid content in Angus and Angus × Simmental muscle tissues^15^. Furthermore, INSIG1/2(/) mice developed pulmonary lipotoxicity because of the accumulation of lipids^36^. The transduction of exogenous INSIG1 significantly reduced cell growth and lipid synthesis^37^. Lipid phosphate phosphatase-related protein type 1 (PLPPR1) extensively participates in lipid-related pathways and was previously referred to as plasticity related gene (PRG), indicating its vital role in meat tenderness. Thus, we considered both INSIG1 and PLPPR1 as lipid deposition-related genes.

From the transcriptome data, a total of 16,705 genes were expressed in the muscle tissue, which helped extensively expand the genetic information pool in duck. Consistent with the phenotypic findings that Cherry Valley Pekin ducks grew faster in 3 weeks, but deposited less lipid in 6 weeks than the native Pekin ducks, functional analysis of DEGs between the two breeds also yielded similar results because the main pathways in which the DEGs at 6 weeks of age were involved included lipid deposition-related categories such as PPAR signalling pathway and adipocytokine signalling pathway, in addition to the muscle growth categories such as cytoskeletal protein binding and muscle tissue development at 3 weeks of age. Hence, DEGs overlapping in the BD3 versus BD6 and CD3 versus CD6 (ACSBG2, FOXO3, and MAT1A) were more likely related to breast growth process involving muscle growth and lipid deposition. The DEGs between the two breeds (ALDOA and TNNT2) at 3 weeks of age were more likely associated with muscle growth, whereas those (ADORA1 and ACSL1) at 6 weeks of age were more likely related to lipid deposition. Functional clustering helped clearly distinguish the 7 DEGs as follows.

Acyl-CoA synthetase bubblegum family member 2 (ACSBG2) has been reported to be significantly associated with fat deposition in chicken by causing the activation of fatty acids^38^. The expression profile of ACSBG2 was similar to that of ENPP3, FABP5, and ADGRG1, suggesting that ACSBG2 was very likely to be a positive lipid deposition regulator. Forkhead box O3 (FOXO3) is a key factor associated with muscle energy homeostasis that controls the glycolytic and lipolytic flow in the FOXO pathway. Many studies have described the roles of FOXO3; for example, the overexpression of FOXO3 reduced muscle mass, FOXO3 expression caused muscle wasting disorders, and FOXO3 inhibited vascular smooth muscle cell proliferation^39–41^. In the present study, FOXO3 was expressed considerably higher in BD than CD at 3 and 6 weeks of age; furthermore, FOXO3 expression increased in BD, but decreased in CD from 3 to 6 weeks of age. The expression profiles validated the repressor role of FOXO3 in muscle growth. The catalytic subunit of methionine adenosyltransferase (MAT1A) catalyses the biosynthesis of *S*-adenosylmethionine (SAM) and provides the substrate for methylation. DNA methylation was shown to be related to muscle growth and lipogenesis^42^, and MAT1A was detected in human and porcine muscles as a DEG involved in muscle growth^43, 44^. In the present study, MAT1A was remarkably expressed in CD than in BD and showed marked reduced expression with growth. MAT1A was detected as a DEG not only for BD3 vs. BD6 and CD3 vs. CD6 but also for BD3 vs. CD3. Moreover, MAT1A level was almost around the critical point of Z(Fst) and Tajima’s *D*. Identification of MAT1A would help in not only explaining the mechanism of muscle growth but also validating the importance of methylation in growth.

Among aldolase A (ALDOA) and troponin T2 (TNNT2) that regulated muscle growth in BD3 vs. CD3, ALDOA was found to be essential for various cellular functions, including cell shape and mobility regulation, muscle contraction, actin filament organization, adenosine triphosphate (ATP) generation, and muscle maintenance^45^. Accumulation of ALDOA during muscle growth was a coordinated process occurring progressively in either intrauterine life or *in vitro* myogenesis^46, 47^. Similar effects of ALDOA have been reported in several other animals (human, rat, pig, sheep, and horse)^48–52^. In the present study, the mRNA expression level of ALDOA increased by nearly five-fold from 3 to 6 weeks of age in CD, whereas no obvious change was observed in BD. Another DEG product, TNNT2, serves as a binding subunit of troponins, which are key proteins regulated by the changes in the intracellular Ca^2+^ concentration and function in striated muscle contraction^53^. TNNT2 was also reported to be correlated with myofibrillogenesis since its depletion disrupted the formation of thin and thick filaments and Z-bodies in the muscle fibre^54^. In the present study, TNNT2 levels decreased from 3 to 6 weeks of age in both the breeds; this result was consistent with those of previous studies in chicken and turkey, suggesting the conservation of TNNT2 function in muscle growth^55, 56^.

Among adenosine A1 receptor (ADORA1) and acyl-CoA synthetase-isoform 1 (ACSL1) that regulate lipid deposition in BD6 vs. CD6, the expression of ADORA1 was observed to be significantly high in BD at 6 weeks of age. The main functions of ADORA1 include the release of neurotransmitters, repair of neuronal damages, beating rhythm of heart, metabolism of lipids, and regulation of renal functions^57^. ADORA1 was also reported to be vital for lipid deposition by conducting transcriptome profiling in pig^58^. ACSL1 is essential for the entire process of lipid metabolism, from biosynthesis to degradation via the conversion of free long-chain fatty acids to fatty acyl-CoA esters^59^. In the present study as well, ACSL1 expression was considerably higher in BD, especially in BD6, showing similar tendencies and functions as those of ENPP3, FABP5, ADGRG1, and ACSBG2.

In conclusion, we screened 23 candidate genes, of which 9 (ENPP3, FABP5, ADGRG1, NGEF, OPN3, FLT1, PODXL, KY, and NEU2) had strong selection signal and were significantly differentially expressed, 7 (TTN, TNNI2, CDH2, MEF2A, PCSK1, INSIG1, and PLPPR1) showed strong selection signal, and 7 (ACSBG2, FOXO3, MAT1A, ALDOA, TNNT2, ADORA1, and ACSL1) showed significantly different expression between the two breeds. Of these, 11 genes (PODXL, KY, NEU2, TTN, TNNI2, CDH2, MEF2A, PCSK1, FOXO3, ALDOA, and TNNT2) were identified to regulate muscle growth, and 10 genes (ENPP3, FABP5, ADGRG1, NGEF, OPN3, INSIG1, PLPPR1, ACSBG2, ADORA1, and ACSL1) were identified to be involved in lipid deposition in duck. FLT1 and MAT1A might have a more complicated regulatory profile covering muscle growth and lipid deposition in Pekin ducks. These results suggest the phenotypic differences between the fat-rich native Pekin and lean-type Cherry Valley Pekin ducks and would provide the basis for further studies on the mechanisms underlying muscle growth and lipid deposition in duck breast muscle.

## Methods

### Animals and sampling

The BD and CD were obtained from two nucleus flocks from Golden Star Duck Co., LTD., Beijing, where all the ducks received routine immunization and were reared under healthy identical conditions. A total of 80 individuals (40 BD and 40 CD), with equal number of males and females, were slaughtered to collect tissue samples from breast muscle at 3 and 6 weeks of age (n = 20). All the samples were immediately stored in liquid nitrogen. Ducks used for WGS were sorted in different lines for different pedigrees to avoid genealogical effects. All procedures for animal care were approved by the Animal Welfare Committee of the State Key Laboratory for Agro-Biotechnology of the China Agricultural University (approval number, XK257). Further, all experiments were conducted in accordance with approved relevant guidelines and regulations.

### Phenotype measurement

The water content in the breast muscle was measured following a previously described method^60^. The IMF was measured using the Soxhlet extraction method. Measurements for each group were performed using three technical replicates and 20 individuals as biological replicates.

Samples of breast muscle stored at −80 °C were embedded in optimum cutting temperature compound and dissected along the horizontal axis into 19–20 nm thick pieces. The sections were stained with haematoxylin and eosin and viewed and imaged using a microscope (×200; Nikon, Tokyo, Japan); three fields of view selected randomly were saved for statistical analysis of the density and diameter of the muscle fibre was determined using equations (1, 2).

1 $$d=\frac{N}{S}$$

2 $$D=2{(\frac{Si}{50\pi })}^{0.5}$$

where, d = density of muscle fibre, N = number of muscle fibres, S = area of the field of view, D = muscle fibre diameter, Si = cross-section area of 50 muscle fibres.

### Genome sequencing and SNP identification

Genomic DNA from nine BD and CD individuals each was extracted from the breast samples by using a standard phenol/chloroform extraction procedure. The quality and integrity of the extracted DNA was checked using NanoDrop 2000 and agarose gel electrophoresis. Paired-end sequencing profiles, with an insert size of 380 bp, were constructed according to the manufacturer’s instructions for TureSeq on the Illumina Hiseq 4000 platform. Sequencing and base calling were performed according to the standard Illumina protocols. Adapter and low-quality reads (reads with unidentified nucleotides (N), ≥10%; reads aligned to the adapter, >10 nt, mismatches allowed, ≤10%; phred quality of bases, <30) were removed from the raw data by using an in-house perl script. The genomic reads were aligned to the duck reference genome (*Anas_platyrhynchos*.*BGI_duck_1*.*0*) by using Burrows-Wheeler algorithm. The alignment outputs (BAM files) were sorted, and PCR duplicates generated during library construction were removed using SAMtools. Genomic variants, including SNPs and indels, were called using genome analysis toolkit. The genomic variants were annotated, with as much detail as possible, by using Python HTseq and an in-house Python script. Sequencing reads of 18 *Anas_platyrhynchos* could be achieved in Sequence Read Archive accession PRJNA358807.

### Heterozygosity analysis

Principal components analysis (PCA) was used to determine the population differences. The *Anas platyrhynchos* genome was assembled using 78,488 scaffolds, most of which were very short for an exact test, especially when very few SNPs were present in one window. Short scaffolds, especially those with less number of SNPs, showed higher Z(Fst) values in our study, which was unreliable and imprecise, indicating the necessity of longer scaffolds. In the present study, 1,592 scaffolds that were larger than 60,000 bp and accounted for up to 95.4% of the whole genome information were sorted for downstream analysis. Neutrality test (Tajima’s *D*) and fixation Z(Fst) were conducted using perl scripts with a sliding window of 40 kb and a step size of 20 kb along the 1,592 scaffolds. The regions were putatively regarded as positively selected sites when they were overlapped by two parameters with extremely high Z(Fst) values (top 5% level) and an extremely low Tajima’s *D* value (top 5% level).

### Functional analysis of PSGs

Functional enrichment analysis was performed to identify the significantly enriched terms and genes in the GO or KEGG pathways. All the ontology functions were confirmed using a combination of results obtained from the analyses by using NCBI (https://www.ncbi.nlm.nih.gov/gene/?term=) databases. Gene interaction diagrams were constructed using the applications in Cytoscape 3.4.0 (http://www.cytoscape.org/) as per the instructions.

### RNA extraction and sequencing

Two breast muscle tissues from each group at the two time points (BD3, BD6, CD3, and CD6) were selected randomly for RNA sequencing. Total RNA was extracted using Trizol reagent (Invitrogen, USA) following the manufacturer’s instructions. RNA-seq libraries were prepared using the Illumina mRNA-seq Prep Kit (Illumina Inc., San Diego, CA) and detailed sequencing was performed using Illumina Hiseq 2000 platform with 100-bp paired-end reads. Clean reads were obtained from raw data after eliminating the sequences with adapters or poly-N and low-quality reads through an in-house perl script. Q20 (false discovery rate, ≤1%), Q30 (false discovery rate, ≤0.1%), and GC content were calculated. Reference genome and gene model annotation files were downloaded from Ensembl (ftp://ftp.ensembl.org/pub/release-74/fasta/anas_platyrhynchos/dna/;ftp://ftp.ensembl.org/pub/release-74/gtf/anas_platyrhynchos/). The paired-end clean reads were aligned to the reference genome by using TopHat v2.0.9. Transcriptome data were deposited in Gene Expression Omnibus (accession number, GSE92920).

### Analysis and annotation of differentially expressed genes

The expression levels of genes were evaluated using reads mapped to the exons. RPKM were used to integrate the multiple gene expression levels^61^. HTSeq analyzer was used to measure the gene expression levels by using the Python framework. A gene with RPKM of less than 0.1 was considered to be not expressed^62^.

DEseq under R was adopted to analyse the DEGs by using read counts of certain genes as replicates^63^. In the present study, the evaluation criteria were significantly different when the padj was <0.05 and fold change was >2 or <0.5. After the gene IDs were transformed to those for humans, DAVID database (http://david.abcc.ncifcrf.gov/) was used for the annotation and clustering of DEGs.

### Quantitative real-time PCR validation of RNA-seq

Eight male ducks from each group were selected randomly for qRT-PCR analysis. Eight genes, including four (MYOG, CAV3, IGFBP5, and TNNI1) that showed similar differential expression patterns, and four (ACSL1, MSTN, CD36, and FHC3) that showed different expression patterns between the breeds, were selected for the validation of the differences in expression as revealed by RNA-seq by using qRT-PCR. The primers were designed considering the basic principles for qRT-PCR primer designing (see Supplementary Table S8). The standard curve was prepared using calibrated samples with five-fold dilutions. All the experiments for each sample were performed in triplicate. The reaction mixture (Real Master Mix SYBR Green I; TIANGEN, China) contained 1 μL of cDNA, 10 μL of SYBR Green mix, 8 μL of ddH_2_O, and 0.5 μL each of forward and reverse primers (10 μM). The PCR protocol included the following steps: 95 °C for 3 min (1 cycle) and 95 °C for 30 s, 60 °C for 30 s, and 72 °C for 30 s (40 cycles). The relative gene expression levels were normalized by comparison with the levels of GAPDH (*Anas platyrhynchos*). The 2^−ΔΔ^Ct method was used to evaluate the relative gene expression levels. All the experiments were performed using CFX96TM Real-Time System (Bio Rad, Hercules, CA, USA).

### Statistical analysis

All the experiments were performed at least thrice or until reproducible results were obtained. Data are presented as means ± standard error. The differences in the values were evaluated using Duncan’s multiple comparison with a Bonferroni justification by using SAS 9.2. The differences were considered significant at *p* < 0.05 and highly significant at <0.01.

## Electronic supplementary material

Supplementary Files
