# Supplementary material for: Integrating genome and transcriptome profiling for elucidating the mechanism of muscle growth and lipid deposition in Pekin ducks
Source: Sci Rep. 2017 Jun 19;7:3837. doi: 10.1038/s41598-017-04178-7 (PMC5476626; doi:10.1038/s41598-017-04178-7)
Supplement: Supplementary file 1 — Supplementary Files [file 41598_2017_4178_MOESM1_ESM.pdf]

Integrating genome and transcriptome profiling for elucidating the mechanism of muscle growth and lipid deposition in pekin ducks

Liyuan Wang[1], Xiangxiang Li[2], Jun Ma[1], Yawen Zhang[1], Hao Zhang[1,*]

[1]National Engineering Laboratory for Animal Breeding, China Agricultural University, Beijing, 100094, People's Republic of China

[2]Beijing Zoo, Beijing, 100044, People's Republic of China

[*]Corresponding author: zhanghao827@163.com.

Supplementary Files

Table…… 2-20

Table S1 2

Table S2 3

Table S3 4-6

Table S4 7

Table S5 8

Table S6 9-11

Table S7 12-19

Table S8 20

Figure…… 21-25

Fig. S1 21

Fig. S2 22

Fig. S3 23

Fig. S4 24

Fig. S5 25

Table S1. Whole genome sequencing data summary

Name	Data_size (Gb)	Q30(%)	Read(M)	Total mapped rate	Unique mapped rate	snp_count	Transitions	Transversions	ts/tv
**CD1**	7.38	96.22	24.6	90.88%	75.14%	1654400	1136812	519017	2.19
**CD11**	6.98	96.41	23.27	89.97%	74.45%	1660914	1143108	519401	2.2
**CD21**	7.16	96.53	23.87	89.32%	73.76%	1692258	1163074	530719	2.19
**CD22**	7.22	96.37	24.07	90.52%	75.09%	1618953	1112053	508318	2.19
**CD28**	7.5	95.27	25	90.37%	74.12%	1721015	1182504	540170	2.19
**CD30**	7.56	94.26	25.2	91.25%	74.09%	1653456	1135968	519057	2.19
**CD32**	6.91	94.83	23.03	91.16%	73.53%	1769649	1217495	554045	2.2
**CD33**	7.52	94.48	25.07	89.75%	72.33%	1808478	1244871	565659	2.2
**CD5**	7.84	96.37	26.13	88.87%	73.34%	2001187	1377651	625622	2.2
**PD1**	7.11	94.46	23.7	90.09%	72.93%	1622718	1115058	509161	2.19
**PD23**	8.86	94.57	29.53	90.07%	72.84%	1812808	1246163	568736	2.19
**PD27**	6.8	94.93	22.67	92.54%	75.29%	1647473	1129732	519218	2.18
**PD29**	7.389	93.35	24.63	91.83%	74.39%	1613048	1107035	507329	2.18
**PD30**	7.38	93.25	24.6	88.28%	70.46%	1729548	1190946	540295	2.2
**PD35**	7.32	94.53	24.4	91.08%	73.77%	1724562	1187199	538933	2.2
**PD4**	7.63	93.64	25.43	90.10%	72.50%	1655858	1140470	516915	2.21
**PD6**	6.76	94.71	22.53	90.00%	72.79%	1727850	1189817	539828	2.2
**PD7**	7.78	94.03	25.93	89.80%	72.97%	1686928	1162704	525843	2.21

Table S2. The SNP distribution in WGS data

Location	Number of SNPs	Percents (%)
Promoter	159,277	3.786
Exon	75,489	1.794
Intergenic	1,956,351	46.505
Intron	2,015,674	47.915
In total	4,206,791	100

Table S3. 206 Positively Selected Genes Screened by Z(Fst) and Tajima’D

Gene Symble	Gene Description	Ensembl ID	Z(Fst)	Tajima'D
**ABHD17B**	Abhydrolase domain-containing protein FAM108B1(ABHD17B)	ENSAPLG00000015189	1.720992	0.023357
**ACOT12**	Acyl-coenzyme A thioesterase 12	ENSAPLG00000005659	1.62897	0.952112
**ACTL7A**	Actin-like protein 7A(ACTL7A)	ENSAPLG00000015520	1.00129	-1.26449
**ADGRA3**	Putative G-protein coupled receptor 132/adhesion G protein-coupled receptor A3	ENSAPLG00000009086	0.927988	1.06656
**ADGRG1**	Adhesion G-protein coupled receptor G1	ENSAPLG00000004555	0.866402	0.34135
**ADGRG5**	Putative G-protein coupled receptor 114	ENSAPLG00000005105	1.033072	0.431343
**ALDH1A1**	Retinal dehydrogenase 1	ENSAPLG00000004796	1.340922	0.532389
**AMMECR1L**	AMMECR1-like protein	ENSAPLG00000009785	1.096388	0.020554
**ANAPC13**	Anaphase-promoting complex subunit 13	ENSAPLG00000005486	0.91139	0.829722
**APTX**	Aprataxin	ENSAPLG00000015645	1.00129	-0.54517
**ARFIP2**	Arfaptin-2	ENSAPLG00000009223	1.278334	0.569397
**ARHGAP15**	Rho GTPase-activating protein 15	ENSAPLG00000008021	0.849965	0.946363
**ARL2BP**	ADP-ribosylation factor-like protein 2-binding protein	ENSAPLG00000012932	1.423807	0.661077
**ARSJ**	Arylsulfatase J	ENSAPLG00000008292	0.899316	0.646206
**ATG16L1**	Autophagy-related protein 16-1	ENSAPLG00000013322	1.42649	0.324203
**ATP9B**	Putative phospholipid-transporting ATPase IIB	ENSAPLG00000013079	0.938982	0.268392
**B4GALT1**	Beta-1,4-galactosyltransferase 1	ENSAPLG00000016180	1.00129	-1.08195
**BRSK2**	BR serine/threonine-protein kinase 2	ENSAPLG00000009397	1.876733	0.578484
**CACYBP**	Calcyclin-binding protein	ENSAPLG00000003474	0.920247	0.65618
**CARNMT1**	Carnosine N-methyltransferase 1	ENSAPLG00000014643	1.780918	0.839717
**CAST**	Calpastatin	ENSAPLG00000006179	1.489972	1.0417
**CCDC102A**	Coiled-coil domain-containing protein 102A	ENSAPLG00000005216	0.891736	0.315639
**CDC42SE2**	CDC42 small effector protein 2	ENSAPLG00000005896	0.967515	0.511765
**CDH2**	Cadherin-2	ENSAPLG00000004203	0.927093	-0.2829
**CDK2AP1**	Cyclin-dependent kinase 2-associated protein 1	ENSAPLG00000016031	1.014977	1.04597
**CDK7**	Cell division protein kinase 7	ENSAPLG00000005769	1.425553	-0.15003
**CEP63**	Centrosomal protein of 63 kDa	ENSAPLG00000005488	0.91139	0.829722
**CIT**	Citron Rho-interacting kinase	ENSAPLG00000009657	1.780369	0.827418
**CITED2**	Cbp/p300-interacting transactivator 2	ENSAPLG00000001420	0.895483	0.48714
**CPNE2**	Copine-2	ENSAPLG00000013415	0.925139	0.34135
**CTRL**	Chymotrypsin-like protease CTRL-1	ENSAPLG00000008866	1.010012	-0.63174
**CWC15**	Protein CWC15-like protein A (cwc15-a)	ENSAPLG00000003221	1.214443	0.582193
**DAB2**	Disabled homolog 2	ENSAPLG00000009262	0.919547	-1.06539
**DARS2**	Aspartyl-tRNA synthetase, mitochondrial	ENSAPLG00000009344	0.917308	0.898943
**DCHS1**	Protocadherin-16	ENSAPLG00000010011	1.057333	-0.30604
**DERL1**	Derlin-1	ENSAPLG00000011042	1.033205	0.945422
**DFNB59**	Pejvakin	ENSAPLG00000012766	0.961566	0.177478
**DGKD**	Diacylglycerol kinase delta	ENSAPLG00000012041	1.750793	1.02807
**DGKQ**	Diacylglycerol kinase theta	ENSAPLG00000016272	1.871217	0
**DHRS12**	Dehydrogenase/reductase SDR family member 12(DHRS12)	ENSAPLG00000007313	2.271036	0.161541
**DHX29**	ATP-dependent RNA helicase DHX29	ENSAPLG00000009145	0.865901	0.261639
**DNAJA1**	DnaJ-like protein subfamily A member 1	ENSAPLG00000015760	1.00129	-0.10852
**DOK4**	Docking protein 4	ENSAPLG00000005258	1.033072	0.34135
**DSP**	Desmoplakin	ENSAPLG00000003818	1.238944	0.720496
**DUS3L**	tRNA-dihydrouridine synthase 3-like	ENSAPLG00000004636	2.470186	0.367629
**EDC4**	Enhancer of mRNA-decapping protein 4	ENSAPLG00000009238	0.964473	-0.12122
**EFHD1**	EF-hand domain-containing protein D1	ENSAPLG00000000594	1.14565	0.25345
**EGFLAM**	Pikachurin/EGF like, fibronectin type III and laminin G domains	ENSAPLG00000010255	0.959086	0.644046
**ENDOD1**	Endonuclease domain-containing 1 protein	ENSAPLG00000003433	1.203217	0.582193
**ENPP1**	Ectonucleotide pyrophosphatase/phosphodiesterase family member 1	ENSAPLG00000009457	0.840257	-2.0924
**ENPP3**	Ectonucleotide pyrophosphatase/phosphodiesterase family member 3	ENSAPLG00000009807	1.92062	-1.4781
**EPHB1**	Ephrin type-B receptor 1	ENSAPLG00000005830	0.811793	1.0807
**ERAP1**	Endoplasmic reticulum aminopeptidase 1(Erap1)	ENSAPLG00000006509	1.292955	0.953976
**ESM1**	Endothelial cell-specific molecule 1	ENSAPLG00000004722	0.945517	0.715846
**F10**	Coagulation factor X	ENSAPLG00000002945	1.195976	0.789509
**FABP5**	Fatty acid-binding protein, epidermal	ENSAPLG00000006597	1.685899	1.158337
**FAM192A**	NEFA-interacting nuclear protein NIP30	ENSAPLG00000013298	1.2066	1.06086
**FAM83A**	Protein FAM83A	ENSAPLG00000010979	0.883134	1.00094
**FKBP7**	Peptidyl-prolyl cis-trans isomerase FKBP7	ENSAPLG00000013044	1.083927	0.059435
**FLT1**	Vascular endothelial growth factor receptor 1/Fms related tyrosine kinase 1	ENSAPLG00000006689	2.329034	0.648954
**FOXI3**	Forkhead box protein I1	ENSAPLG00000001701	0.860666	0.672675
**GALNT7**	N-acetylgalactosaminyltransferase 7	ENSAPLG00000003409	0.832724	0.682728
**GAS2L3**	Protein FAM98A	ENSAPLG00000013956	1.559584	0.354261
**GDA**	Guanine deaminase	ENSAPLG00000015274	0.994974	0.67771
**GIGYF2**	PERQ amino acid-rich with GYF domain-containing protein 2	ENSAPLG00000014162	0.879092	0.246058
**GPI**	Glucose-6-phosphate isomerase	ENSAPLG00000010107	0.864915	0.734558
**GPR17**	Putative G-protein coupled receptor 97	ENSAPLG00000000907	0.801899	-0.61206
**GPX8**	Putative glutathione peroxidase 8-B	ENSAPLG00000009098	0.813779	0.491033
**GRXCR1**	Glutaredoxin domain-containing cysteine-rich protein 1	ENSAPLG00000009269	0.80999	0.413644
**GZMA**	Granzyme A	ENSAPLG00000004213	0.946069	0.511765
**HECA**	Headcase protein-like protein	ENSAPLG00000003922	1.271568	0.648954
**HMCN1**	Hemicentin-1	ENSAPLG00000011251	1.726164	0
**HMGB2**	High mobility group protein B2	ENSAPLG00000003284	0.930026	0.302707
**HPX**	Hemopexin	ENSAPLG00000008972	0.938767	-0.30604
**HS6ST1**	Heparan-sulfate 6-O-sulfotransferase 1	ENSAPLG00000007002	0.998333	-0.58604
**IKBKAP**	Elongator complex protein 1(IKBKAP)	ENSAPLG00000014409	0.903219	-1.08195
**ILK**	Integrin-linked protein kinase	ENSAPLG00000009542	1.018462	0.569397
**INSIG1**	Insulin-induced gene 1 protein	ENSAPLG00000007430	0.952497	0.878962
**IWS1**	Protein IWS1 homolog	ENSAPLG00000011485	2.122438	0.585745
**JAG2**	Protein jagged-2	ENSAPLG00000004118	0.996992	1.0076
**KATNBL1**	Katanin regulatory subunit B1 like 1	ENSAPLG00000004713	0.994259	0.918893
**KCNK18**	Potassium channel subfamily K member 18	ENSAPLG00000010704	0.968188	0.789509
**KCNMB2**	Calcium-activated potassium channel subunit beta-2	ENSAPLG00000013434	1.405168	-0.35665
**KY**	Kyphoscoliosis peptidase	ENSAPLG00000005689	0.91139	0.488917
**LCMT2**	Leucine carboxyl methyltransferase 2	ENSAPLG00000010069	0.878237	0.5433

**LIMS2**	LIM and senescent cell antigen-like-containing domain protein 2	ENSAPLG00000010258	0.912341	0.581031
**LIPH**	Lipase member H	ENSAPLG00000005090	1.793805	0.829722
**LOC101789877**	F-box/WD repeat-containing protein 7	ENSAPLG00000009463	1.072061	0.411715
**LOC101801055**	UPF0600 protein	ENSAPLG00000011429	0.952916	-0.45944
**LOC101801209**	Metastasis suppressor protein 1	ENSAPLG00000007146	1.250483	0.48062
**LOC101801727**	Selenium-binding protein 1-A	ENSAPLG00000010148	1.526292	0.966677
**LOC101803127**	Transmembrane protein ENSP00000382582	ENSP00000382582	0.8659	-1.73768
**LOC101804425**	Tubulin alpha-2 chain	ENSAPLG00000007864	1.07018	1.02994
**LSP1**	Lymphocyte-specific protein 1	ENSAPLG00000011632	1.180634	0.33643
**LTBP1**	Latent-transforming growth factor beta-binding protein 1(Ltbp1)	ENSAPLG00000012621	1.724828	1.05862
**MAP3K13**	Mitogen-activated protein kinase kinase kinase 13	ENSAPLG00000004597	0.809557	0.773791
**MED23**	Mediator of RNA polymerase II transcription subunit 23	ENSAPLG00000010330	1.3967	-1.0614
**MEF2A**	Myocyte-specific enhancer factor 2A	ENSAPLG00000007352	1.290035	-1.38531
**MKLN1**	Muskelin	ENSAPLG00000003364	2.414354	0.519611
**MOB2**	Mps one binder kinase activator-like 2	ENSAPLG00000009842	1.873221	0.33643
**MPHOSPH9**	M-phase phosphoprotein 9	ENSAPLG00000016075	1.014977	1.04597
**MRPL23**	39S ribosomal protein L23, mitochondrial	ENSAPLG00000012955	1.025438	1.06745
**MRPL53**	39S ribosomal protein L53, mitochondrial	ENSAPLG00000006944	1.099508	0.858337
**MRPS14**	28S ribosomal protein S14, mitochondrial	ENSAPLG00000003382	0.916052	-0.22621
**MRPS36**	28S ribosomal protein S36, mitochondrial	ENSAPLG00000005421	1.425553	0.562068
**MTMR12**	Myotubularin-related protein 12	ENSAPLG00000013094	2.751323	-1.17276
**MTSS1**	Metastasis suppressor protein 1(MTSS1)	ENSAPLG00000015070	2.861473	1.00324
**MTX3**	Metaxin-3	ENSAPLG00000006896	0.960027	0.409689
**MYO7B**	Unconventional myosin-VIIb(MYO7B)	ENSAPLG00000010464	2.124102	-1.84836
**NCSTN**	Nicastrin	ENSAPLG00000014723	0.945516	-0.31438
**NEU2**	Sialidase-2/Neuraminidase 2	ENSAPLG00000013917	1.217374	0.899084
**NGEF**	Ephexin-1	ENSAPLG00000013970	0.820379	0.517968
**NMRK1**	Nicotinamide riboside kinase 1	ENSAPLG00000014721	1.567147	0.839717
**NPR3**	Atrial natriuretic peptide clearance receptor	ENSAPLG00000016025	1.16917	-1.56262
**NUDT14**	Uridine diphosphate glucose pyrophosphatase	ENSAPLG00000003998	1.208367	0.451212
**NUP155**	Nuclear pore complex protein Nup155	ENSAPLG00000008857	0.807714	-0.99801
**NUTF2**	Nuclear transport factor 2	ENSAPLG00000009210	0.842292	-0.12122
**OPN3**	Opsin-3	ENSAPLG00000014871	0.977454	0.473539
**OSBPL6**	Oxysterol-binding protein-related protein 6	ENSAPLG00000011717	0.897008	0.92766
**OSTF1**	Osteoclast-stimulating factor 1	ENSAPLG00000014884	1.151684	0.839717
**PACS2**	Phosphofurin acidic cluster sorting protein 2	ENSAPLG00000015577	0.909023	0.337753
**PARP12**	Poly [ADP-ribose] polymerase 12	ENSAPLG00000006979	1.441757	0.35781
**PAX5**	Paired box protein Pax-5	ENSAPLG00000010841	2.459154	1.03426
**PCSK2**	Neuroendocrine convertase 1	ENSAPLG00000014969	1.207289	1.05459
**PDE1C**	Calcium/calmodulin-dependent 3',5'-cyclic nucleotide phosphodiesterase 1C(Pde1c)	ENSAPLG00000008225	1.513352	1.02144
**PDZD2**	PDZ domain-containing protein 2	ENSAPLG00000012765	1.031388	-0.17407
**PDZD8**	PDZ domain-containing protein 8	ENSAPLG00000011185	1.242359	0.553777
**PIGM**	GPI mannosyltransferase 1	ENSAPLG00000014870	0.915037	1.06729
**PIK3CB**	Phosphatidylinositol-4,5-bisphosphate 3-kinase catalytic subunit beta isoform	ENSAPLG00000003554	0.928103	0.502938
**PIK3R1**	Phosphatidylinositol 3-kinase regulatory subunit alpha(PIK3R1)	ENSAPLG00000004772	3.189838	0.477607
**PLLP**	Plasmolipin	ENSAPLG00000012794	1.423807	0.661077
**PLPP1**	Lipid phosphate phosphohydrolase 1	ENSAPLG00000010771	0.910714	0.661077
**PLPPR1**	Lipid phosphate phosphatase-related protein type 1	ENSAPLG00000006758	6.501825	-0.15928
**PODXL**	Podocalyxin-like protein 1	ENSAPLG00000003276	2.572196	0.343329
**POGZ**	Pogo transposable element with ZNF domain	ENSAPLG00000009926	1.971557	1.05862
**POLR2C**	DNA-directed RNA polymerase II subunit RPB3	ENSAPLG00000005385	0.891736	0.830357
**POLR2D**	DNA-directed RNA polymerase II subunit RPB4	ENSAPLG00000009812	0.802122	-0.20438
**POMT1**	Protein O-mannosyl-transferase 1(POMT1)	ENSAPLG00000014827	1.277645	0.994116
**PPP1R17**	G-substrate(human:Protein phosphatase 1 regulatory subunit 17 PPP1R17)	ENSAPLG00000009013	1.515938	1.02144
**PRKAB1**	5'-AMP-activated protein kinase subunit beta-1	ENSAPLG00000009490	1.561006	0.744369
**PRRC2B**	Uncharacterized protein KIAA0515(PRRC2B)	ENSAPLG00000014308	1.223569	0.994116
**PRUNE2**	Protein prune-like protein 2(PRUNE2)	ENSAPLG00000008078	3.081234	0.807237
**PSKH1**	Serine/threonine-protein kinase H1	ENSAPLG00000009622	1.010012	-0.96243
**PSMB4**	Proteasome subunit beta type-4	ENSAPLG00000009850	1.610869	1.05862
**R0JER9**	Putative polypeptide N-acetylgalactosaminyltransferase 17	ENSAPLG00000003852	1.452865	0.860163
**R0KC87**	LysM and putative peptidoglycan-binding domain-containing protein 4(LYSMD4)	ENSAPLG00000001341	0.979691	0.511886
**RABGAP1**	Rab GTPase-activating protein 1	ENSAPLG00000006937	0.886993	0.300774
**RABGAP1L**	RAB GTPase-activating protein 1-like	ENSAPLG00000005959	0.789425	0.39963
**RASGRP3**	Ras guanyl-releasing protein 3	ENSAPLG00000012309	1.785497	0.267915
**RC3H1**	Roquin	ENSAPLG00000005341	0.897465	0.025934
**RIOK1**	Serine/threonine-protein kinase RIO1	ENSAPLG00000004196	1.182153	0.594772
**RNF139**	RING finger protein 139	ENSAPLG00000011464	1.534825	-0.29306
**RSPRY1**	RING finger and SPRY domain-containing protein 1	ENSAPLG00000012937	1.423807	1.06086
**SAG**	S-antigen visual arrestin	ENSAPLG00000012828	1.131999	0.485883
**SAP130**	Histone deacetylase complex subunit SAP130	ENSAPLG00000009195	2.048463	-0.14763
**SAP30**	Histone deacetylase complex subunit SAP30	ENSAPLG00000003274	0.930026	0.337753
**SBNO1**	Protein strawberry notch-like protein 1	ENSAPLG00000015674	1.253036	1.04597
**SEMA6A**	Semaphorin-6A	ENSAPLG00000014502	1.145154	0.960773
**SERINC5**	Serine incorporator 5	ENSAPLG00000005798	0.939759	-0.73227
**SESN3**	Sestrin-3	ENSAPLG00000003440	1.378998	0.313691
**SLC18A2**	Synaptic vesicular amine transporter	ENSAPLG00000010707	0.968188	0.789509
**SLC30A5**	Zinc transporter 5(SLC30A5)	ENSAPLG00000005422	0.914603	0.790575
**SLC30A9**	Zinc transporter 9	ENSAPLG00000007998	1.045405	0.720469
**SLCO4C1**	Solute carrier organic anion transporter family 4C1	ENSAPLG00000009323	0.827448	-1.83175
**SMAD2**	Mothers against decapentaplegic-like protein 2(SMAD2)	ENSAPLG00000008095	1.054894	0.360367
**SMU1**	WD40 repeat-containing protein SMU1	ENSAPLG00000015994	1.00129	-1.08195
**SSBP2**	Single-stranded DNA-binding protein 2	ENSAPLG00000005451	1.61046	-0.44535
**SSR1**	Translocon-associated protein subunit alpha	ENSAPLG00000004473	0.921062	0.594772
**SUB1**	Activated RNA polymerase II transcriptional coactivator p15	ENSAPLG00000015978	1.143366	-1.773
**TARS**	Threonyl-tRNA synthetase, cytoplasmic	ENSAPLG00000016069	1.175753	0.744369
**TATDN1**	Putative deoxyribonuclease TATDN1	ENSAPLG00000006883	1.526054	-0.29306
**TBC1D31**	WD repeat-containing protein 67/TBC1 domain family member 31	ENSAPLG00000010985	0.961318	0.88246
**TBXAS1**	Thromboxane-A synthase	ENSAPLG00000006656	1.236563	0.268392
**THAP11**	THAP domain-containing protein 11	ENSAPLG00000002084	0.842292	-0.12122

**THBS1**	Thrombospondin-1	ENSAPLG00000003908	0.928733	0.787054
**THBS4**	Thrombospondin-4	ENSAPLG00000006149	0.944433	-0.04061
**TLE1**	Transducin-like enhancer protein 1	ENSAPLG00000012402	0.822217	1.05859
**TMC1**	Transmembrane channel-like protein 1	ENSAPLG00000004487	1.288731	0.755648
**TMEM161B**	Transmembrane protein 161B	ENSAPLG00000010679	1.0541	0.376283
**TMEM2**	Transmembrane protein 2(TMEM2)	ENSAPLG00000014669	0.919547	0.371385
**TMEM245**	Transmembrane protein C9orf5(TMEM245)	ENSAPLG00000009827	1.520344	0.905477
**TMEM33**	Transmembrane protein 33	ENSAPLG00000007860	0.93721	0.720469
**TMEM41A**	TMEM41A	ENSAPLG00000005037	0.793805	0.773791
**TMEM65**	Transmembrane protein 65	ENSAPLG00000006782	2.067027	0.919496
**TNN**	Tenascin-N	ENSAPLG00000003018	0.920247	0.65618
**TNNI2**	Troponin I, fast skeletal muscle	ENSAPLG00000011264	1.031603	0.498567
**TNNT3**	Troponin T, fast skeletal muscle isoforms	ENSAPLG00000011980	1.941518	0.132705
**TNR**	Tenascin-R	ENSAPLG00000001323	1.041721	0.783862
**TRIM3**	Tripartite motif-containing protein 3	ENSAPLG00000009137	1.177121	0.411715
**TRPM6**	Transient receptor potential cation channel subfamily M member 6	ENSAPLG00000014165	2.035566	0.773225
**TSC22D1**	TSC22 domain family protein 1	ENSAPLG00000005147	1.411148	0.996987
**TSNAXIP1**	Translin-associated factor X-interacting protein 1	ENSAPLG00000008665	0.842292	-0.31438
**TTN**	Titin	ENSAPLG00000013370	1.719764	0.265324
**TUBA8**	Tubulin alpha-8 chain	ENSAPLG00000007746	1.07018	1.02994
**TXLNB**	Beta-taxilin	ENSAPLG00000003963	1.183457	0.648954
**UGGT1**	UDP-glucose:glycoprotein glucosyltransferase 1	ENSAPLG00000007044	2.139839	-0.58604
**USP18**	ubiquitin specific peptidase 18	ENSAPLG00000007508	1.07018	1.02994
**WDR33**	WD repeat-containing protein 33	ENSAPLG00000009868	1.077551	-1.14134
**WDR36**	WD repeat-containing protein 36(WDR36)	ENSAPLG00000012829	0.919547	-2.35375
**WDR70**	WD repeat-containing protein 70	ENSAPLG00000010164	1.489918	-1.3312
**ZFR**	Zinc finger RNA-binding protein	ENSAPLG00000015627	0.968718	-1.51923
**ZHX1**	Zinc fingers and homeoboxes protein 1	ENSAPLG00000010954	1.459071	0.977132
**ZHX2**	Zinc fingers and homeoboxes protein 2	ENSAPLG00000001368	0.880491	0
**ZMAT3**	Zinc finger matrin-type protein 3	ENSAPLG00000013292	1.174525	-2.03632
**ZNF462**	Zinc finger protein 462	ENSAPLG00000012931	0.872628	0.905077
**ZNF516**	Zinc finger protein 516	ENSAPLG00000015083	1.863955	0.745986

Table S4. Obtained clean data from raw data

	Raw reads	Clean reads	clean bases	Error rate(%)	Q20(%)	Q30(%)	GC content(%)
BD3_1	56654722	51168536	5.12G	0.05	95	85.83	51.88
BD3_2	54338258	50378540	5.04G	0.06	94.495	85.485	50.96
BD6_1	59421916	55284218	5.52G	0.05	94.485	85.37	51.345
BD6_2	49388830	46056976	4.6G	0.05	94.585	85.57	50.025
CD3_1	48743458	45645158	4.56G	0.05	95.025	86.67	48.455
CD3_2	56927652	53163828	5.32G	0.05	94.925	86.4	49.15
CD6_1	50149094	46515126	4.66G	0.05	94.615	85.765	50.37
CD6_2	53365442	49470316	4.94G	0.06	94.49	85.43	51.005

BD: Native Pekin ducks, CD: Cherry Valley Pekin ducks

Table S5. Statistics of Mapping of Groups

Sample name	BD3_1	BD3_2	BD6_1	BD6_2	CD3_1	CD3_2	CD6_1	CD6_2
**Total reads**	51168536	50378540	55284218	46056976	45645158	53163828	46515126	49470316
**Total mapped**	32862320 (64.22%)	32275711 (64.07%)	35400104 (64.03%)	30624644 (66.49%)	31925364 (69.94%)	37009182 (69.61%)	30811837 (66.24%)	31869481 (64.42%)
**Multiple mapped**	1312355 (2.56%)	922465 (1.83%)	1342930 (2.43%)	797113 (1.73%)	693967 (1.52%)	967580 (1.82%)	852141 (1.83%)	1111494 (2.25%)
**Uniquely mapped**	31549965 (61.66%)	31353246 (62.24%)	34057174 (61.6%)	29827531 (64.76%)	31231397 (68.42%)	36041602 (67.79%)	29959696 (64.41%)	30757987 (62.17%)
**Read-1**	15818736 (30.91%)	15710036 (31.18%)	17087607 (30.91%)	14945369 (32.45%)	15640492 (34.27%)	18047764 (33.95%)	15023565 (32.3%)	15418719 (31.17%)
**Read-2**	15731229 (30.74%)	15643210 (31.05%)	16969567 (30.7%)	14882162 (32.31%)	15590905 (34.16%)	17993838 (33.85%)	14936131 (32.11%)	15339268 (31.01%)
**Reads map to '+'**	15579976 (30.45%)	15578250 (30.92%)	16790321 (30.37%)	14744491 (32.01%)	15510714 (33.98%)	17870489 (33.61%)	14831660 (31.89%)	15175465 (30.68%)
**Reads map to '-'**	15969989 (31.21%)	15774996 (31.31%)	17266853 (31.23%)	15083040 (32.75%)	15720683 (34.44%)	18171113 (34.18%)	15128036 (32.52%)	15582522 (31.5%)
**Non-splice reads**	20285278 (39.64%)	22302413 (44.27%)	22210729 (40.18%)	20049916 (43.53%)	21495719 (47.09%)	24345701 (45.79%)	20001209 (43%)	20070618 (40.57%)
**Splice reads**	11264687 (22.01%)	9050833 (17.97%)	11846445 (21.43%)	9777615 (21.23%)	9735678 (21.33%)	11695901 (22%)	9958487 (21.41%)	10687369 (21.6%)

BD: Native Pekin ducks, CD: Cherry Valley Pekin ducks

Table S6 Function Analysis of DEGs in BD3VSBD6 and CD3VSCD6

BD3VSBD6	Term	Count	PValue
GO_CC	plasma membrane part	43	5.33E-07
GO_CC	plasma membrane	57	1.12E-05
GO_CC	integral to plasma membrane	25	1.22E-04
GO_CC	intrinsic to plasma membrane	25	1.72E-04
GO_CC	apical junction complex	6	0.001973
GO_CC	apicolateral plasma membrane	6	0.002249
GO_CC	occluding junction	5	0.0042
GO_CC	tight junction	5	0.0042
GO_CC	neuromuscular junction	3	0.016483
GO_CC	guanylate cyclase complex, soluble	2	0.017914
GO_CC	cell junction	11	0.018263
GO_CC	Golgi apparatus	15	0.024548
GO_CC	proteinaceous extracellular matrix	8	0.025433
GO_CC	cell-cell junction	6	0.028466
GO_CC	basement membrane	4	0.033158
GO_CC	extracellular matrix	8	0.036124
GO_CC	cell surface	8	0.037581
GO_MF	GTPase regulator activity	13	1.86E-04
GO_MF	nucleoside-triphosphatase regulator activity	13	2.29E-04
GO_MF	Ras guanyl-nucleotide exchange factor activity	6	9.69E-04
GO_MF	small GTPase regulator activity	9	0.002546
GO_MF	transmembrane receptor protein tyrosine kinase activity	5	0.002649
GO_MF	Rho guanyl-nucleotide exchange factor activity	5	0.003798
GO_MF	guanyl-nucleotide exchange factor activity	6	0.010141
GO_MF	GTPase activator activity	7	0.011956
GO_MF	growth factor binding	5	0.012881
GO_MF	enzyme activator activity	8	0.025871
GO_MF	mRNA 5'-UTR binding	2	0.034067
GO_MF	nucleotide binding	28	0.040857
GO_BP	cell adhesion	17	4.95E-04
GO_BP	biological adhesion	17	5.03E-04
GO_BP	muscle system process	8	7.75E-04
GO_BP	vasodilation	4	0.001562
GO_BP	regulation of cell motion	8	0.001733
GO_BP	regulation of Rho protein signal transduction	6	0.001929
GO_BP	membrane organization	11	0.002203
GO_BP	regulation of angiogenesis	5	0.002415
GO_BP	muscle contraction	7	0.002507
GO_BP	regulation of hydrolase activity	10	0.003255
GO_BP	regulation of muscle contraction	5	0.003922
GO_BP	regulation of cell migration	7	0.004098
GO_BP	epithelium development	8	0.004282
GO_BP	blood circulation	7	0.0065
GO_BP	circulatory system process	7	0.0065
GO_BP	nitric oxide mediated signal transduction	3	0.00673
GO_BP	regulation of locomotion	7	0.007552
GO_BP	intracellular signaling cascade	21	0.007777
GO_BP	angiogenesis	6	0.010562
GO_BP	regulation of G-protein coupled receptor protein signaling pathway	4	0.010639
GO_BP	regulation of Ras protein signal transduction	7	0.011446
GO_BP	muscle organ development	7	0.011697
GO_BP	regulation of blood vessel size	4	0.011815
GO_BP	regulation of tube size	4	0.011815
GO_BP	regulation of cell shape	4	0.01243
GO_BP	morphogenesis of an epithelium	5	0.01278
GO_BP	muscle cell development	4	0.013713
GO_BP	membrane invagination	7	0.014136
GO_BP	endocytosis	7	0.014136
GO_BP	vascular process in circulatory system	4	0.01507
GO_BP	tissue morphogenesis	6	0.02276
GO_BP	MAPKKK cascade	6	0.024738
GO_BP	regulation of small GTPase mediated signal transduction	7	0.025654
GO_BP	tube morphogenesis	5	0.027142
GO_BP	regulation of apoptosis	14	0.02749
GO_BP	skin development	3	0.027523
GO_BP	regulation of programmed cell death	14	0.029478
GO_BP	regulation of cell death	14	0.03025
GO_BP	vesicle-mediated transport	11	0.033648
GO_BP	regulation of cell adhesion	5	0.03449
GO_BP	negative regulation of Rho protein signal transduction	2	0.035304
GO_BP	muscle fiber development	3	0.038965
GO_BP	blood vessel morphogenesis	6	0.040977
GO_BP	respiratory gaseous exchange	3	0.041021
GO_BP	regulation of lipase activity	4	0.043023
GO_BP	cholesterol transport	3	0.043117
GO_BP	sterol transport	3	0.043117
GO_BP	regulation of smooth muscle contraction	3	0.045252
GO_BP	tube development	6	0.047549
GO_BP	negative regulation of signal transduction	6	0.048316
KEGG_PATHWAY	Glycine, serine and threonine metabolism	4	0.005296
KEGG_PATHWAY	ECM-receptor interaction	5	0.015837
KEGG_PATHWAY	Focal adhesion	7	0.02795
KEGG_PATHWAY	PPAR signaling pathway	4	0.045319
KEGG_PATHWAY	Endocytosis	6	0.061014
KEGG_PATHWAY	Vascular smooth muscle contraction	4	0.139978
KEGG_PATHWAY	Riboflavin metabolism	2	0.170562
KEGG_PATHWAY	Long-term depression	3	0.190466
KEGG_PATHWAY	Cell adhesion molecules (CAMs)	4	0.196784
KEGG_PATHWAY	Tight junction	4	0.202761

**KEGG_PATHWAY**	Selenoamino acid metabolism	2	0.262278
**KEGG_PATHWAY**	Hematopoietic cell lineage	3	0.263384
**KEGG_PATHWAY**	Gap junction	3	0.276375
**KEGG_PATHWAY**	Fructose and mannose metabolism	2	0.328406
**KEGG_PATHWAY**	Calcium signaling pathway	4	0.334897
**KEGG_PATHWAY**	Cytokine-cytokine receptor interaction	5	0.361882
**KEGG_PATHWAY**	ABC transporters	2	0.402919
**KEGG_PATHWAY**	mTOR signaling pathway	2	0.456623
**KEGG_PATHWAY**	Inositol phosphate metabolism	2	0.46929
**KEGG_PATHWAY**	Vibrio cholerae infection	2	0.481665
**KEGG_PATHWAY**	Purine metabolism	3	0.534643
**KEGG_PATHWAY**	Adipocytokine signaling pathway	2	0.544828
**KEGG_PATHWAY**	Long-term potentiation	2	0.550179
**KEGG_PATHWAY**	Viral myocarditis	2	0.565866
**KEGG_PATHWAY**	Hypertrophic cardiomyopathy (HCM)	2	0.632244
**KEGG_PATHWAY**	Dilated cardiomyopathy	2	0.661583
**KEGG_PATHWAY**	Melanogenesis	2	0.688618
**KEGG_PATHWAY**	Leukocyte transendothelial migration	2	0.751749
**KEGG_PATHWAY**	Neuroactive ligand-receptor interaction	3	0.805778
**KEGG_PATHWAY**	Pathways in cancer	3	0.902173
**KEGG_PATHWAY**	Regulation of actin cytoskeleton	2	0.922991
**KEGG_PATHWAY**	MAPK signaling pathway	2	0.95928
**CD3VSCD6**			
**GO_CC**	extracellular region part	28	2.96E-06
**GO_CC**	extracellular region	44	3.22E-06
**GO_CC**	extracellular matrix	14	8.45E-05
**GO_CC**	extracellular space	19	3.73E-04
**GO_CC**	proteinaceous extracellular matrix	11	0.002331
**GO_CC**	platelet alpha granule lumen	4	0.009539
**GO_CC**	cytoplasmic membrane-bounded vesicle lumen	4	0.011579
**GO_CC**	vesicle lumen	4	0.013068
**GO_CC**	basolateral plasma membrane	7	0.02206
**GO_CC**	platelet alpha granule	4	0.022093
**GO_CC**	adherens junction	6	0.02562
**GO_CC**	anchoring junction	6	0.037654
**GO_MF**	receptor tyrosine kinase binding	3	0.002884
**GO_MF**	actin binding	10	0.006993
**GO_MF**	carbohydrate binding	9	0.031516
**GO_MF**	cytoskeletal protein binding	11	0.0374
**GO_MF**	phosphatase activity	7	0.045644
**GO_MF**	vitamin binding	5	0.046639
**GO_BP**	regulation of programmed cell death	20	3.30E-04
**GO_BP**	regulation of cell death	20	3.46E-04
**GO_BP**	response to wounding	15	6.64E-04
**GO_BP**	regulation of apoptosis	19	8.05E-04
**GO_BP**	response to hormone stimulus	12	9.31E-04
**GO_BP**	skeletal system development	11	0.001137
**GO_BP**	tube development	9	0.001412
**GO_BP**	response to endogenous stimulus	12	0.002044
**GO_BP**	negative regulation of programmed cell death	11	0.002715
**GO_BP**	negative regulation of cell death	11	0.00277
**GO_BP**	organic acid biosynthetic process	7	0.004114
**GO_BP**	carboxylic acid biosynthetic process	7	0.004114
**GO_BP**	regeneration	5	0.004589
**GO_BP**	ossification	6	0.005347
**GO_BP**	regulation of inflammatory response	5	0.006464
**GO_BP**	muscle cell differentiation	6	0.006613
**GO_BP**	bone development	6	0.007078
**GO_BP**	negative regulation of apoptosis	10	0.007869
**GO_BP**	tube morphogenesis	6	0.008077
**GO_BP**	positive regulation of apoptosis	11	0.009441
**GO_BP**	positive regulation of programmed cell death	11	0.009884
**GO_BP**	positive regulation of cell death	11	0.010188
**GO_BP**	DNA replication initiation	3	0.010368
**GO_BP**	response to organic substance	15	0.010677
**GO_BP**	striated muscle cell differentiation	5	0.010746
**GO_BP**	wound healing	7	0.011064
**GO_BP**	negative regulation of hydrolase activity	4	0.011407
**GO_BP**	fatty acid metabolic process	7	0.013037
**GO_BP**	induction of apoptosis	9	0.013168
**GO_BP**	induction of programmed cell death	9	0.013397
**GO_BP**	lipid transport	6	0.013788
**GO_BP**	cellular component morphogenesis	10	0.01577
**GO_BP**	negative regulation of response to stimulus	5	0.016533
**GO_BP**	muscle organ development	7	0.017334
**GO_BP**	cardiac muscle tissue development	4	0.018116
**GO_BP**	lipid localization	6	0.018824
**GO_BP**	ureteric bud morphogenesis	3	0.019213
**GO_BP**	branching involved in ureteric bud morphogenesis	3	0.019213
**GO_BP**	regulation of response to external stimulus	6	0.019767
**GO_BP**	regulation of I-kappaB kinase/NF-kappaB cascade	5	0.020662
**GO_BP**	regulation of cell proliferation	15	0.021344
**GO_BP**	vitamin transport	3	0.022667
**GO_BP**	negative regulation of cell proliferation	9	0.02514
**GO_BP**	branching morphogenesis of a tube	4	0.025572
**GO_BP**	positive regulation of gene expression	12	0.026732
**GO_BP**	epithelial tube morphogenesis	4	0.027657
**GO_BP**	fatty acid transport	3	0.03027
**GO_BP**	tissue morphogenesis	6	0.031569
**GO_BP**	morphogenesis of a branching structure	4	0.035644
**GO_BP**	response to metal ion	5	0.037501
**GO_BP**	succinyl-CoA metabolic process	2	0.038467
**GO_BP**	tissue regeneration	3	0.038732

**GO_BP**	inflammatory response	8	0.04006
**GO_BP**	positive regulation of nucleobase, nucleoside, nucleotide and nucleic acid metabolic process	12	0.0416
**GO_BP**	response to hypoxia	5	0.042169
**GO_BP**	response to nutrient levels	6	0.043804
**GO_BP**	ureteric bud development	3	0.045596
**GO_BP**	negative regulation of defense response	3	0.047977
**GO_BP**	regulation of MAP kinase activity	5	0.049232
**GO_BP**	response to oxygen levels	5	0.049232
**GO_BP**	positive regulation of transcription	11	0.049244
**KEGG_PATHWAY**	Propanoate metabolism	4	0.006362
**KEGG_PATHWAY**	Steroid biosynthesis	3	0.017148
**KEGG_PATHWAY**	PPAR signaling pathway	4	0.049229
**KEGG_PATHWAY**	Glycine, serine and threonine metabolism	3	0.052694
**KEGG_PATHWAY**	Axon guidance	5	0.068125
**KEGG_PATHWAY**	DNA replication	3	0.068744
**KEGG_PATHWAY**	Pyruvate metabolism	3	0.082586
**KEGG_PATHWAY**	Cell cycle	4	0.188634
**KEGG_PATHWAY**	Biosynthesis of unsaturated fatty acids	2	0.233609
**KEGG_PATHWAY**	Selenoamino acid metabolism	2	0.269892
**KEGG_PATHWAY**	Pyrimidine metabolism	3	0.31619
**KEGG_PATHWAY**	Cysteine and methionine metabolism	2	0.337462
**KEGG_PATHWAY**	Fatty acid metabolism	2	0.384066
**KEGG_PATHWAY**	Cytokine-cytokine receptor interaction	5	0.385694
**KEGG_PATHWAY**	Starch and sucrose metabolism	2	0.398873
**KEGG_PATHWAY**	Lysine degradation	2	0.413329
**KEGG_PATHWAY**	Intestinal immune network for IgA production	2	0.447989
**KEGG_PATHWAY**	Arginine and proline metabolism	2	0.47426
**KEGG_PATHWAY**	Natural killer cell mediated cytotoxicity	3	0.477288
**KEGG_PATHWAY**	Insulin signaling pathway	3	0.485172
**KEGG_PATHWAY**	Glycolysis / Gluconeogenesis	2	0.517308
**KEGG_PATHWAY**	Adipocytokine signaling pathway	2	0.556883
**KEGG_PATHWAY**	Glycerophospholipid metabolism	2	0.56227
**KEGG_PATHWAY**	Complement and coagulation cascades	2	0.567592
**KEGG_PATHWAY**	Renal cell carcinoma	2	0.572851
**KEGG_PATHWAY**	Pancreatic cancer	2	0.58318
**KEGG_PATHWAY**	Chronic myeloid leukemia	2	0.598214
**KEGG_PATHWAY**	ECM-receptor interaction	2	0.64019
**KEGG_PATHWAY**	Hypertrophic cardiomyopathy (HCM)	2	0.644579
**KEGG_PATHWAY**	Hematopoietic cell lineage	2	0.648915
**KEGG_PATHWAY**	ErbB signaling pathway	2	0.653199
**KEGG_PATHWAY**	Chemokine signaling pathway	3	0.663151
**KEGG_PATHWAY**	Dilated cardiomyopathy	2	0.67386
**KEGG_PATHWAY**	Melanogenesis	2	0.700765
**KEGG_PATHWAY**	Focal adhesion	3	0.70195
**KEGG_PATHWAY**	Leukocyte transendothelial migration	2	0.763271
**KEGG_PATHWAY**	Pathways in cancer	4	0.763623
**KEGG_PATHWAY**	Cell adhesion molecules (CAMs)	2	0.800923
**KEGG_PATHWAY**	Tight junction	2	0.805797
**KEGG_PATHWAY**	MAPK signaling pathway	3	0.83852
**KEGG_PATHWAY**	Wnt signaling pathway	2	0.842755
**KEGG_PATHWAY**	Jak-STAT signaling pathway	2	0.85039
**KEGG_PATHWAY**	Huntington's disease	2	0.890491
**KEGG_PATHWAY**	Endocytosis	2	0.89584

Table S7. DEGs Statistic in BD3 VS BD6, CD3 VS CD6, BD3 VS CD3 and BD6 VS CD6

Differential expression genes between 3-week and 6-week old in Native Pekin ducks(BD3, BD6)

Gene_id	readcount_BD6	readcount_BD3	log2FoldChange	pval	padj
**ENSAPLG00000000572**	348.9938621	86.80689008	2.0073	0.00050259	0.047011
**ENSAPLG00000000583**	3352.851018	768.2521792	2.1257	2.17E-05	0.0051937
**ENSAPLG00000000788**	94.16466562	12.1282718	2.9568	0.00029066	0.031665
**ENSAPLG00000000798**	336.9918506	52.86562114	2.6723	4.73E-06	0.00161
**ENSAPLG00000000900**	774.4123574	159.7161319	2.2776	2.95E-05	0.0064878
**ENSAPLG00000000930**	43.76659554	195.0645286	-2.156	0.00049354	0.0467
**ENSAPLG00000001030**	330.7552119	80.98165306	2.0301	0.00042867	0.042697
**ENSAPLG00000001042**	22.12033705	119.5568815	-2.4343	0.00049341	0.0467
**ENSAPLG00000001269**	264.1263082	44.06668578	2.5835	1.79E-05	0.0043534
**ENSAPLG00000001671**	132.4688532	25.33429875	2.3865	0.00025981	0.030103
**ENSAPLG00000001679**	155.1326959	19.7228853	2.9756	1.44E-05	0.0037041
**ENSAPLG00000001980**	148.2207005	19.21018479	2.9478	9.01E-05	0.014216
**ENSAPLG00000001983**	636.1234279	166.5493185	1.9334	0.00038767	0.03907
**ENSAPLG00000002133**	24.64430745	150.2408231	-2.608	6.59E-05	0.011454
**ENSAPLG00000002229**	44.78417971	218.3153261	-2.2854	0.00011754	0.016991
**ENSAPLG00000002397**	80.94660443	482.2266255	-2.5747	4.42E-06	0.001537
**ENSAPLG00000002412**	536.1667551	135.4617918	1.9848	0.00042585	0.042665
**ENSAPLG00000002538**	2185.983263	397.9768042	2.4575	1.23E-06	0.00067545
**ENSAPLG00000002574**	456.2356395	42.81000981	3.4138	1.89E-06	0.00084307
**ENSAPLG00000002687**	2359.027105	38694.97217	-4.0359	2.71E-14	1.54E-10
**ENSAPLG00000002862**	2568.439558	685.6668493	1.9053	8.93E-05	0.014216
**ENSAPLG00000002906**	1350.792242	7374.641374	-2.4488	8.26E-07	0.00050231
**ENSAPLG00000002989**	22.12033705	146.0802543	-2.7233	4.35E-05	0.0085172
**ENSAPLG00000003010**	165.0672794	34.04597721	2.2775	0.00044329	0.043642
**ENSAPLG00000003098**	151.6956405	1977.450438	-3.7044	5.06E-12	1.72E-08
**ENSAPLG00000003217**	77.98572293	415.1143285	-2.4122	1.38E-05	0.00362
**ENSAPLG00000003276**	756.3132786	216.4564892	1.8049	0.00054177	0.047545
**ENSAPLG00000003502**	423.9022934	88.89700193	2.2535	9.22E-05	0.014407
**ENSAPLG00000003585**	2989.730918	560.7494842	2.4146	1.40E-06	0.00069765
**ENSAPLG00000003622**	685.7521751	211.7896537	1.6951	0.00044251	0.043642
**ENSAPLG00000003824**	230.2795726	1167.738616	-2.3423	7.12E-06	0.0022896
**ENSAPLG00000003972**	20.58659903	244.9018938	-3.5724	4.12E-08	4.38E-05
**ENSAPLG00000004359**	20.55433942	114.4952749	-2.4778	0.00037796	0.038318
**ENSAPLG00000004522**	1825.835238	516.2988681	1.8223	0.00029374	0.031665
**ENSAPLG00000004523**	868.0931288	187.3172598	2.2124	3.80E-05	0.0078069
**ENSAPLG00000004541**	440.3842008	5543.018057	-3.6538	3.76E-12	1.60E-08
**ENSAPLG00000004555**	714.7031695	129.3681736	2.4659	5.25E-06	0.0017204
**ENSAPLG00000004563**	7130.645905	838.1636607	3.0887	3.06E-09	4.00E-06
**ENSAPLG00000004628**	0	143.1567065		5.35E-15	4.56E-11
**ENSAPLG00000004634**	685.9085653	107.1122814	2.6789	8.66E-07	0.00050875
**ENSAPLG00000004669**	133.5285127	10.46360357	3.6737	1.38E-06	0.00069765
**ENSAPLG00000004686**	168.0008091	29.12398158	2.5282	0.00010844	0.016061
**ENSAPLG00000004727**	565.9487791	2181.298301	-1.9464	0.000125	0.017595
**ENSAPLG00000004830**	14.6178529	146.6082026	-3.3262	3.17E-06	0.0012864
**ENSAPLG00000004908**	444.624934	60.9576873	2.8667	1.42E-06	0.00069765
**ENSAPLG00000004909**	4.117319807	73.47209292	-4.1574	8.78E-06	0.0026716
**ENSAPLG00000004913**	575.7494163	108.3188068	2.4102	1.54E-05	0.0039229
**ENSAPLG00000005101**	775.2980906	175.935167	2.1397	9.93E-05	0.015095
**ENSAPLG00000005130**	74.28777812	3.381690578	4.4573	4.75E-05	0.0090915
**ENSAPLG00000005167**	5.968746131	170.4938603	-4.8361	2.19E-10	4.15E-07
**ENSAPLG00000005280**	552.4670158	83.56701405	2.7249	1.53E-06	0.00072453
**ENSAPLG00000005391**	110.0532718	717.5288473	-2.7048	0.0005134	0.047098
**ENSAPLG00000005396**	423.003932	107.2388481	1.9798	0.00048457	0.046676
**ENSAPLG00000005523**	2354.699409	657.7648178	1.8399	0.00028304	0.031665
**ENSAPLG00000005535**	746.3281821	141.1953649	2.4021	9.49E-06	0.0027407
**ENSAPLG00000005651**	35.91416347	530.6261903	-3.8851	2.97E-05	0.0064878
**ENSAPLG00000005660**	2929.203272	9503.101816	-1.6979	0.00052613	0.047098
**ENSAPLG00000005933**	233.0216399	51.99728251	2.164	0.00021934	0.026318
**ENSAPLG00000005934**	1328.103143	381.1251124	1.801	0.00037386	0.038199
**ENSAPLG00000005951**	1175.939767	3793.068367	-1.6896	0.00052813	0.047098
**ENSAPLG00000006115**	257.6140564	47.00548136	2.4543	3.99E-05	0.0080348
**ENSAPLG00000006127**	341.5383611	87.51375927	1.9645	0.00052134	0.047098
**ENSAPLG00000006318**	52.40588663	453.1136615	-3.1121	4.39E-08	4.40E-05
**ENSAPLG00000006380**	2527.600867	495.8211665	2.3499	5.11E-06	0.0017055
**ENSAPLG00000006405**	715.9732054	2488.248858	-1.7972	0.00029192	0.031665
**ENSAPLG00000006412**	273.264217	57.94688269	2.2375	0.00013305	0.018276
**ENSAPLG00000006597**	11486.3731	1863.80367	2.6236	1.28E-07	0.00010411
**ENSAPLG00000006664**	391.1474769	1389.455464	-1.8287	0.00020003	0.025051
**ENSAPLG00000006689**	1061.358181	165.5915195	2.6802	5.29E-07	0.00034668
**ENSAPLG00000006699**	1010.004951	263.8198122	1.9367	0.00025675	0.029952
**ENSAPLG00000006824**	172.5325961	18.50111208	3.2212	2.01E-06	0.00085698
**ENSAPLG00000007034**	1638.142718	395.184407	2.0515	0.00016179	0.021302
**ENSAPLG00000007114**	46.54373503	307.0988057	-2.722	3.70E-06	0.0014208
**ENSAPLG00000007141**	63.46955671	1554.226415	-4.614	4.02E-16	6.84E-12
**ENSAPLG00000007153**	4056.818682	1149.324761	1.8196	0.00016618	0.021605
**ENSAPLG00000007168**	288.3287967	50.65334964	2.509	2.86E-05	0.0064878
**ENSAPLG00000007445**	313.3251473	45.44772492	2.7854	3.30E-06	0.0013089
**ENSAPLG00000007476**	213.8572766	20.32504623	3.3953	3.21E-07	0.00023734
**ENSAPLG00000007608**	114.719005	483.3414869	-2.0749	0.00016025	0.021302
**ENSAPLG00000007778**	92.43246207	496.5846201	-2.4256	1.35E-05	0.0035884
**ENSAPLG00000007797**	18.54652286	102.4739149	-2.466	0.00052935	0.047098
**ENSAPLG00000007866**	575.0186388	53.256162	3.4326	0.00017463	0.022363
**ENSAPLG00000007924**	2630.068939	405.5932761	2.697	1.50E-07	0.00011579
**ENSAPLG00000008004**	407.242269	100.1547316	2.0237	0.00045616	0.044652
**ENSAPLG00000008093**	498.3015739	129.5819971	1.9432	0.0004878	0.046676
**ENSAPLG00000008117**	2347.353976	668.2761917	1.8125	0.00016259	0.021302
**ENSAPLG00000008411**	446.618027	119.5765364	1.9011	0.00050113	0.047011
**ENSAPLG00000008432**	13623.49717	2973.603687	2.1958	4.16E-06	0.0015075

**ENSAPLG00000008462**	585.115955	101.6033727	2.5258	0.00021394	0.026028
**ENSAPLG00000008491**	34125.46037	119393.7824	-1.8068	0.00014392	0.01961
**ENSAPLG00000008567**	224.6579619	2238.673518	-3.3168	1.27E-07	0.00010411
**ENSAPLG00000008590**	6696.583155	1349.95734	2.3105	3.95E-06	0.0014642
**ENSAPLG00000008624**	291.3689209	56.44368391	2.368	9.35E-05	0.014483
**ENSAPLG00000008924**	622.223007	139.0506954	2.1618	4.94E-05	0.0093433
**ENSAPLG00000009003**	5.010773354	149.9724418	-4.9035	8.50E-10	1.34E-06
**ENSAPLG00000009157**	494.7432005	108.6525865	2.187	5.75E-05	0.010198
**ENSAPLG00000009268**	379.9666793	76.96069529	2.3037	3.59E-05	0.007498
**ENSAPLG00000009277**	2310.920846	505.8942292	2.1916	9.71E-05	0.014901
**ENSAPLG00000009614**	1715.69153	457.6932401	1.9063	0.0003057	0.032542
**ENSAPLG00000009777**	952.2979544	182.1421308	2.3863	4.39E-06	0.001537
**ENSAPLG00000009807**	477.4842497	122.6069959	1.9614	0.00050807	0.047029
**ENSAPLG00000009829**	958.8859756	165.800936	2.5319	1.17E-05	0.0031674
**ENSAPLG00000009911**	27.16337001	168.1026679	-2.6296	5.40E-05	0.0097884
**ENSAPLG00000010094**	361.2090627	87.99376055	2.0374	0.0005299	0.047098
**ENSAPLG00000010141**	17.84171916	119.9845287	-2.7495	8.03E-05	0.013538
**ENSAPLG00000010204**	708.9770594	148.421296	2.256	4.43E-05	0.0085767
**ENSAPLG00000010246**	474.2652913	86.76978381	2.4504	1.03E-05	0.0028778
**ENSAPLG00000010278**	594.8394448	150.9607366	1.9783	7.95E-05	0.013538
**ENSAPLG00000010424**	111.9741252	420.4487234	-1.9088	0.00032878	0.034691
**ENSAPLG00000010515**	509.2312976	138.0950999	1.8827	0.00046565	0.045319
**ENSAPLG00000010532**	673.574142	176.4107612	1.9329	0.00034985	0.036556
**ENSAPLG00000010847**	8052.76683	2249.909566	1.8396	0.00012819	0.017886
**ENSAPLG00000010852**	8.649106772	343.5622407	-5.3119	8.77E-06	0.0026716
**ENSAPLG00000010917**	465.0502349	93.6946344	2.3113	5.17E-05	0.0094619
**ENSAPLG00000010951**	8389.939667	1761.795393	2.2516	1.74E-05	0.0043062
**ENSAPLG00000010971**	1029.860112	279.4998813	1.8815	0.00021231	0.026014
**ENSAPLG00000011011**	1040.681146	184.2322426	2.4979	1.63E-06	0.00074955
**ENSAPLG00000011156**	211.9364232	31.35590796	2.7568	8.36E-05	0.013688
**ENSAPLG00000011160**	394.6911268	99.88414683	1.9824	0.00012454	0.017595
**ENSAPLG00000011184**	1183.464638	88.63302774	3.739	0.00015995	0.021302
**ENSAPLG00000011190**	704.4775321	168.5303151	2.0635	0.00010778	0.016061
**ENSAPLG00000011374**	141.7533366	19.7228853	2.8454	4.01E-05	0.0080348
**ENSAPLG00000011482**	491.9134527	105.3778077	2.2228	5.50E-05	0.0098668
**ENSAPLG00000011742**	153.4523833	30.09702847	2.3501	0.00024913	0.029467
**ENSAPLG00000011749**	30.95809367	606.0489608	-4.291	8.66E-10	1.34E-06
**ENSAPLG00000011759**	102.5556955	15.66922829	2.7104	0.00048648	0.046676
**ENSAPLG00000011871**	120.9184763	17.20953335	2.8128	0.00017014	0.021954
**ENSAPLG00000011914**	490.9134046	120.6980084	2.0241	0.00053094	0.047098
**ENSAPLG00000011941**	80.33857958	7.241178914	3.4718	5.01E-05	0.0093433
**ENSAPLG00000012198**	1807.462641	20810.80648	-3.5253	9.93E-12	2.82E-08
**ENSAPLG00000012212**	114.9819898	12.60606955	3.1892	3.61E-05	0.007498
**ENSAPLG00000012276**	12.76642658	163.0366542	-3.6748	0.00012149	0.017388
**ENSAPLG00000012291**	155.8697592	33.14273583	2.2336	0.00054435	0.047545
**ENSAPLG00000012345**	437.7108433	77.23128003	2.5027	2.70E-05	0.0062949
**ENSAPLG00000012368**	116.7639891	18.20003162	2.6816	0.00015586	0.021068
**ENSAPLG00000012754**	1033.612043	3406.912321	-1.7208	0.00035682	0.036833
**ENSAPLG00000012815**	664.5701794	92.59722435	2.8434	4.81E-07	0.00032785
**ENSAPLG00000012966**	657.3363052	151.2836755	2.1194	5.05E-05	0.0093433
**ENSAPLG00000013023**	229.2591759	43.94452614	2.3832	0.00019868	0.025051
**ENSAPLG00000013272**	1101.570594	130.7819119	3.0743	1.08E-07	9.79E-05
**ENSAPLG00000013290**	34.31099839	188.3404573	-2.4566	8.25E-05	0.013688
**ENSAPLG00000013386**	2658.082913	244.655371	3.4416	2.47E-08	3.01E-05
**ENSAPLG00000013559**	2085.938966	619.8483346	1.7507	0.00035409	0.036773
**ENSAPLG00000013669**	538.2138344	114.6916471	2.2304	5.88E-05	0.010322
**ENSAPLG00000013757**	231.6695487	837.6575708	-1.8543	0.00026249	0.030207
**ENSAPLG00000013917**	3.22386626	46.5276836	-3.8512	0.00025517	0.029952
**ENSAPLG00000013920**	497.7258086	133.2102105	1.9016	0.00052453	0.047098
**ENSAPLG00000013970**	31.63554559	255.0666204	-3.0113	9.45E-07	0.00053657
**ENSAPLG00000014093**	3713.835641	1079.481058	1.7826	0.00032996	0.034691
**ENSAPLG00000014181**	13319.68855	2697.852506	2.3037	2.40E-06	0.00099498
**ENSAPLG00000014219**	1148.401192	280.5340964	2.0334	0.00011772	0.016991
**ENSAPLG00000014282**	245.2943566	43.30525894	2.5019	3.48E-05	0.0074078
**ENSAPLG00000014530**	141.6193903	14.18127737	3.32	1.05E-05	0.0028778
**ENSAPLG00000014692**	3117.89314	848.2890776	1.8779	0.00012917	0.017886
**ENSAPLG00000014731**	51.29643146	261.0838226	-2.3476	8.29E-05	0.013688
**ENSAPLG00000015082**	111.0140571	1678.164123	-3.9181	2.05E-11	4.99E-08
**ENSAPLG00000015151**	666.4166979	121.8760648	2.451	3.75E-06	0.0014208
**ENSAPLG00000015253**	733.7335866	195.5118307	1.908	0.00027926	0.031499
**ENSAPLG00000015271**	389.9784104	64.3219265	2.6	8.79E-06	0.0026716
**ENSAPLG00000015363**	226.8817801	994.3496769	-2.1318	3.39E-05	0.0073044
**ENSAPLG00000015453**	181.4299641	40.63043755	2.1588	0.00029322	0.031665
**ENSAPLG00000015509**	485.0168982	107.4679195	2.1741	8.99E-05	0.014216
**ENSAPLG00000015757**	2552.413476	631.5293044	2.0149	4.24E-05	0.0083932
**ENSAPLG00000015867**	309.0865095	1222.541694	-1.9838	8.64E-05	0.014008
**ENSAPLG00000015868**	2680.346408	556.8137566	2.2672	9.01E-06	0.0026716
**ENSAPLG00000016039**	1364.628805	149.6932198	3.1884	0.00021942	0.026318
**ENSAPLG00000016064**	880.2396196	254.0368124	1.7929	0.00030155	0.032302
**ENSAPLG00000016184**	104.6974583	14.28818915	2.8733	0.00023039	0.02744
**ENSAPLG00000016190**	11.10855795	78.5359031	-2.8217	0.00050511	0.047011
**ENSAPLG00000016206**	8.39593769	220.1567116	-4.7127	2.49E-11	5.30E-08
**ENSAPLG00000016231**	2071.740488	467.6504002	2.1473	9.10E-06	0.0026716
**ENSAPLG00000016291**	71.50082295	489.5769197	-2.7755	7.33E-07	0.00046254
**ENSAPLG00000016389**	1166.251292	324.8210396	1.8442	0.00027544	0.031275
**Novel00003**	101.5254831	362.2007603	-1.8349	0.0005337	0.047098
**Novel00088**	19.75766472	117.3969642	-2.5709	0.00029124	0.031665
**Novel00113**	39.57984867	208.7724153	-2.3991	7.19E-05	0.012365
**Novel00165**	2.744879871	48.3865205	-4.1398	0.00011039	0.016209
**Novel00182**	80.77549067	327.707481	-2.0204	0.00037454	0.038199
**Novel00199**	31.40972828	240.3551912	-2.9359	1.93E-06	0.00084307
**Novel00301**	20.58659903	122.0200829	-2.5673	0.00018793	0.023886
**Novel00337**	32.62087014	164.8453405	-2.3372	0.00026478	0.030267

**Novel00382**	548.6926408	111.8052056	2.295	2.93E-05	0.0064878
**Novel00576**	72.41672043	551.2328389	-2.9283	1.09E-07	9.79E-05
**Novel00600**	196.6705654	40.71989796	2.272	0.00020926	0.025827
**Novel00770**	149.4395627	16.65972656	3.1651	9.89E-06	0.0028062
**Novel00948**	230.2395926	29.01706979	2.9882	1.43E-06	0.00069765
**Novel00956**	1.372439936	46.42077182	-5.08	2.52E-05	0.0059573
**Novel01042**	4.56404658	140.9247802	-4.9485	3.74E-08	4.25E-05
**Novel01233**	16.85148677	133.0509446	-2.981	2.91E-05	0.0064878
**Novel01340**	80.76777026	645.9921841	-2.9997	1.74E-05	0.0043062
**Novel01487**	248.0315161	57.22035861	2.1159	0.00028586	0.031665
**Novel01557**	89.27802288	512.4283622	-2.521	0.00010203	0.015379
**Novel01692**	34.40777724	286.2632625	-3.0565	4.31E-07	0.00030551
**Novel01916**	0	80.22022622		1.21E-09	1.72E-06
**Novel02174**	127.0485205	19.05091887	2.7374	0.00020493	0.025478
**Differential expression genes between 3-week and 6-week old in Cherry Valley Pekinducks(CD3, CD6)**					
**Gene_id**	readcount_CD6	readcount_CD3	log2FoldChange	pval	padj
**ENSAPLG00000000701**	453.2759938	1141.908121	-1.333	0.00044095	0.037256
**ENSAPLG00000000874**	372.2332438	140.767856	1.4029	0.00058518	0.044238
**ENSAPLG00000000926**	103.1999724	457.9976287	-2.1499	3.21E-07	0.00014961
**ENSAPLG00000000980**	1434.154886	3773.536452	-1.3957	9.79E-05	0.013078
**ENSAPLG00000001082**	159.4721214	0.890746355	7.4841	6.30E-06	0.001596
**ENSAPLG00000001519**	48.2961495	0.843100526	5.8401	5.58E-08	3.71E-05
**ENSAPLG00000001879**	219.507137	43.36999494	2.3395	0.00026829	0.026347
**ENSAPLG00000001929**	164.94288	34.25538736	2.2676	8.15E-06	0.0019513
**ENSAPLG00000002217**	334.257341	122.2911131	1.4506	0.00055569	0.042768
**ENSAPLG00000002226**	3.244728287	51.11739788	-3.9776	0.00011882	0.015245
**ENSAPLG00000002416**	6042.808785	97.82667349	5.9488	9.20E-41	1.59E-36
**ENSAPLG00000002436**	295.343237	1415.976196	-2.2613	8.71E-05	0.011944
**ENSAPLG00000002488**	249.8239709	29.40392823	3.0868	3.93E-10	6.16E-07
**ENSAPLG00000002602**	38.40349938	154.6697161	-2.0099	6.99E-05	0.01044
**ENSAPLG00000002669**	8.074092109	84.63223857	-3.3898	1.65E-06	0.00059222
**ENSAPLG00000002749**	6.680619388	47.9666642	-2.844	0.000568	0.043511
**ENSAPLG00000002858**	976.0872826	403.8526843	1.2732	0.00066646	0.048583
**ENSAPLG00000002895**	16317.61616	4671.526271	1.8045	0.0001528	0.017688
**ENSAPLG00000002897**	165.0283993	30.26358948	2.4471	2.77E-06	0.0008998
**ENSAPLG00000003004**	4.127596414	181.6893166	-5.46	2.09E-16	1.74E-12
**ENSAPLG00000003145**	406.7430693	1028.114256	-1.3378	0.00055581	0.042768
**ENSAPLG00000003202**	211.6971536	62.30663534	1.7645	0.00014447	0.017173
**ENSAPLG00000003331**	14522.34563	46081.36116	-1.6659	3.02E-06	0.00096496
**ENSAPLG00000003383**	633.760872	1703.630667	-1.4266	0.0001532	0.017688
**ENSAPLG00000003705**	234.294546	858.3640222	-1.8733	1.11E-06	0.00041731
**ENSAPLG00000003784**	66.11448856	1086.820712	-4.039	1.49E-12	3.20E-09
**ENSAPLG00000003961**	18.32894366	123.5485017	-2.7529	3.48E-06	0.0010536
**ENSAPLG00000004048**	5415.997698	1476.537993	1.875	3.96E-07	0.00017054
**ENSAPLG00000004054**	211.0280838	48.01431003	2.1359	2.21E-05	0.0045262
**ENSAPLG00000004098**	308.0228326	50.54361161	2.6074	8.70E-09	9.83E-06
**ENSAPLG00000004145**	356.5428425	106.1955085	1.7474	3.68E-05	0.0066066
**ENSAPLG00000004233**	1186.413599	335.112979	1.8239	1.95E-06	0.00067389
**ENSAPLG00000004284**	40.08371633	144.6001556	-1.851	0.00041842	0.036255
**ENSAPLG00000004359**	54.82836573	418.8079786	-2.9333	3.73E-06	0.0010608
**ENSAPLG00000004477**	48.2961495	181.6561952	-1.9112	0.00012725	0.015689
**ENSAPLG00000004506**	137.2595659	0	Inf	0.00016162	0.018108
**ENSAPLG00000004520**	91.57177236	317.8895521	-1.7956	4.68E-05	0.007986
**ENSAPLG00000004542**	46.53041324	175.7948751	-1.9176	0.00012029	0.015245
**ENSAPLG00000004581**	5603.940403	704.3756019	2.992	1.08E-14	3.12E-11
**ENSAPLG00000004635**	1161.738579	380.6072859	1.6099	0.00012743	0.015689
**ENSAPLG00000004718**	468.3425962	1142.684709	-1.2868	0.00046595	0.038798
**ENSAPLG00000004765**	284.8343388	99.97480844	1.5105	0.00059605	0.044474
**ENSAPLG00000004794**	827.4736875	280.0235335	1.5632	3.95E-05	0.0070212
**ENSAPLG00000004821**	3345.460943	1169.080571	1.5168	0.00027266	0.026402
**ENSAPLG00000004881**	106.7213828	20.43225814	2.3849	4.09E-05	0.0071212
**ENSAPLG00000004896**	17.90385838	111.9118548	-2.644	6.83E-06	0.0016852
**ENSAPLG00000004909**	17.25491272	92.08446801	-2.416	7.87E-05	0.011116
**ENSAPLG00000005167**	20.36129994	104.6429464	-2.3616	4.83E-05	0.0080877
**ENSAPLG00000005304**	8.62745636	205.9849595	-4.5775	1.09E-14	3.12E-11
**ENSAPLG00000005377**	1234.322383	498.3566379	1.3085	0.00059596	0.044474
**ENSAPLG00000005388**	190.7372185	605.8603704	-1.6674	6.02E-05	0.0094363
**ENSAPLG00000005402**	65.41272115	2.155397144	4.9235	9.70E-09	9.83E-06
**ENSAPLG00000005404**	138.6882474	376.5451726	-1.441	0.00053398	0.042413
**ENSAPLG00000005423**	991.5789363	10343.43074	-3.3828	1.01E-09	1.28E-06
**ENSAPLG00000005459**	3643.996086	970.8165418	1.9083	3.72E-07	0.00016866
**ENSAPLG00000005481**	19602.10888	3785.619355	2.3724	4.32E-06	0.0011629
**ENSAPLG00000005489**	445.3578557	1416.000902	-1.6688	1.66E-05	0.0036393
**ENSAPLG00000005505**	151.156556	434.007611	-1.5217	0.00027098	0.026387
**ENSAPLG00000005584**	5973.859552	2121.79977	1.4934	4.78E-05	0.0080789
**ENSAPLG00000005651**	456.483002	5112.726096	-3.4855	6.92E-07	0.00027734
**ENSAPLG00000005712**	1761.427285	650.1272206	1.438	0.00010292	0.013438
**ENSAPLG00000005719**	841.1342269	213.985116	1.9748	0.00015655	0.017751
**ENSAPLG00000005722**	8068.668861	3447.963703	1.2266	0.00070302	0.049865
**ENSAPLG00000005738**	28.3825703	108.2866991	-1.9318	0.00038203	0.033768
**ENSAPLG00000005762**	590.4953113	1458.508046	-1.3045	0.00051004	0.041459
**ENSAPLG00000005792**	0	153.7115737		0.00032402	0.029549
**ENSAPLG00000005881**	1126.84894	384.6353948	1.5507	3.64E-05	0.0065952
**ENSAPLG00000005950**	7087.289477	2649.333442	1.4196	7.03E-05	0.01044
**ENSAPLG00000006033**	65.60388397	212.1725382	-1.6934	0.00042831	0.036474
**ENSAPLG00000006048**	56.17907879	251.3984992	-2.1619	4.50E-06	0.0011924
**ENSAPLG00000006103**	2403.858373	6469.752309	-1.4284	0.00010149	0.013353
**ENSAPLG00000006199**	78.30611499	242.0860461	-1.6283	0.00037779	0.033739
**ENSAPLG00000006249**	35.30717425	0.445373178	6.3088	3.11E-06	0.00097538
**ENSAPLG00000006292**	661.9422174	1711.781023	-1.3707	0.00020408	0.022123
**ENSAPLG00000006311**	13267.14396	36993.74112	-1.4794	8.80E-05	0.011944
**ENSAPLG00000006362**	163.5141985	549.4917497	-1.7487	0.00012536	0.015689

**ENSAPLG00000006380**	1325.655194	468.4515453	1.5007	0.00035925	0.032419
**ENSAPLG00000006430**	608.8267662	117.1444805	2.3777	9.35E-09	9.83E-06
**ENSAPLG00000006677**	1329.206807	511.5551454	1.3776	0.00023915	0.025134
**ENSAPLG00000006683**	14.60630834	126.7945271	-3.1178	7.21E-08	4.29E-05
**ENSAPLG00000006702**	68.93413157	14.66622975	2.2327	0.00057396	0.043773
**ENSAPLG00000006717**	15566.42385	5271.873294	1.562	2.29E-05	0.0045927
**ENSAPLG00000006891**	79.6140684	299.3910227	-1.9109	2.66E-05	0.0050916
**ENSAPLG00000007063**	112.1242351	336.7474616	-1.5866	0.00025368	0.025848
**ENSAPLG00000007148**	221.3131216	74.30992454	1.5745	0.00053644	0.042413
**ENSAPLG00000007168**	178.0500721	45.13492693	1.98	0.00050494	0.041443
**ENSAPLG00000007183**	788.0137531	310.6299423	1.343	0.0005829	0.044238
**ENSAPLG00000007341**	73.2428287	421.6065944	-2.5251	0.00068603	0.049269
**ENSAPLG00000007359**	785.2041892	2605.027091	-1.7302	6.84E-06	0.0016852
**ENSAPLG00000007409**	772.5899718	255.8304444	1.5945	5.72E-05	0.0091296
**ENSAPLG00000007410**	343.8506735	129.8644935	1.4048	0.00064587	0.047371
**ENSAPLG00000007440**	116.7196765	28.34642148	2.0418	0.00014977	0.017681
**ENSAPLG00000007523**	178.1330802	748.3559642	-2.0708	6.58E-05	0.010031
**ENSAPLG00000007626**	3969.288967	1422.50836	1.4804	4.58E-05	0.0078864
**ENSAPLG00000007893**	1972.777321	440.0127516	2.1646	1.47E-08	1.41E-05
**ENSAPLG00000007931**	88.23146266	390.9897339	-2.1478	8.49E-07	0.00032536
**ENSAPLG00000008063**	376.863894	993.7760652	-1.3989	0.00023352	0.024693
**ENSAPLG00000008065**	54.60450535	178.3956454	-1.708	0.00052829	0.042351
**ENSAPLG00000008162**	2095.370206	637.3999455	1.7169	4.58E-06	0.001195
**ENSAPLG00000008175**	31.40343821	117.2967165	-1.9012	0.00049002	0.040605
**ENSAPLG00000008327**	46.583235	0.421550263	6.788	2.57E-05	0.0049797
**ENSAPLG00000008336**	1864.597054	439.625476	2.0845	5.60E-08	3.71E-05
**ENSAPLG00000008363**	526.752745	171.2127302	1.6213	5.86E-05	0.0092651
**ENSAPLG00000008381**	9679.269042	3800.669401	1.3486	0.0002468	0.025472
**ENSAPLG00000008389**	30.03008969	192.8930785	-2.6833	8.80E-05	0.011944
**ENSAPLG00000008403**	3601.39705	11246.11346	-1.6428	4.29E-06	0.0011629
**ENSAPLG00000008717**	7226.784982	954.70207	2.9202	1.18E-07	6.36E-05
**ENSAPLG00000008739**	270.9272866	71.04733849	1.9311	1.16E-05	0.0026983
**ENSAPLG00000008874**	66.51944965	5.058603156	3.717	1.15E-06	0.00042231
**ENSAPLG00000008992**	73.57233257	222.4751019	-1.5964	0.00052367	0.042177
**ENSAPLG00000009016**	1544.910812	548.9499316	1.4928	8.45E-05	0.011751
**ENSAPLG00000009426**	17.66993591	78.11840131	-2.1444	0.00054446	0.042672
**ENSAPLG00000009563**	19.43567216	112.564372	-2.534	1.39E-05	0.0031518
**ENSAPLG00000009580**	3526.358333	1429.605282	1.3026	0.000458	0.038321
**ENSAPLG00000009581**	2796.41014	1048.818748	1.4148	0.00013775	0.016603
**ENSAPLG00000009685**	40.17929773	186.5314772	-2.2149	1.32E-05	0.0030443
**ENSAPLG00000009847**	28.01030677	124.9964736	-2.1579	5.08E-05	0.0084134
**ENSAPLG00000009870**	14233.82142	4392.786453	1.6961	4.22E-06	0.0011629
**ENSAPLG00000010071**	602.322225	1508.404367	-1.3244	0.00050844	0.041459
**ENSAPLG00000010094**	209.1541928	36.64901365	2.5127	2.51E-07	0.00012006
**ENSAPLG00000010133**	189.6430634	39.68789495	2.2565	3.40E-06	0.0010465
**ENSAPLG00000010141**	82.65757178	302.2869665	-1.8707	2.14E-05	0.00444
**ENSAPLG00000010494**	54.64726501	239.6903835	-2.133	0.00020999	0.022481
**ENSAPLG00000010520**	17.95668013	151.0590849	-3.0725	8.17E-08	4.69E-05
**ENSAPLG00000010759**	290.4308652	102.0587368	1.5088	0.00067003	0.048583
**ENSAPLG00000010917**	268.3667128	81.25053794	1.7238	0.00011984	0.015245
**ENSAPLG00000010935**	13.77626196	72.75010026	-2.4008	0.00028345	0.026992
**ENSAPLG00000010999**	1653.272215	8.414444544	7.6182	6.32E-07	0.00025925
**ENSAPLG00000011037**	126.1469929	476.6048209	-1.9177	2.40E-06	0.00079523
**ENSAPLG00000011053**	177.3457935	486.160729	-1.4549	0.00040316	0.035454
**ENSAPLG00000011054**	27.97760921	157.0819395	-2.4892	0.00019566	0.021345
**ENSAPLG00000011178**	18.46728472	90.96275471	-2.3003	0.00025049	0.025699
**ENSAPLG00000011184**	1660.093652	165.667217	3.3249	3.02E-16	1.74E-12
**ENSAPLG00000011263**	14.14852549	83.27955832	-2.5573	0.00015393	0.017688
**ENSAPLG00000011292**	300.6630813	64.38330155	2.2234	8.17E-07	0.00031985
**ENSAPLG00000011300**	306.0256851	61.92546871	2.305	3.95E-07	0.00017054
**ENSAPLG00000011513**	27.69086499	132.5346962	-2.2589	0.00025967	0.026021
**ENSAPLG00000011553**	15885.76035	3020.906004	2.3947	0.00025494	0.025848
**ENSAPLG00000011574**	97.33682606	412.4835714	-2.0833	1.84E-06	0.00064699
**ENSAPLG00000011787**	267.6221858	848.0376355	-1.6639	1.69E-05	0.0036393
**ENSAPLG00000011847**	7444.810735	1541.864029	2.2716	1.04E-09	1.28E-06
**ENSAPLG00000011914**	555.1453774	207.0134175	1.4231	0.00037304	0.033488
**ENSAPLG00000012199**	1316.192651	3123.98709	-1.247	0.000417	0.036255
**ENSAPLG00000012207**	187.1856218	546.3999967	-1.5455	0.00016182	0.018108
**ENSAPLG00000012230**	548.8093295	2223.150198	-2.0182	9.52E-06	0.0022473
**ENSAPLG00000012347**	3538.451884	1443.080366	1.294	0.00042069	0.036255
**ENSAPLG00000012360**	818.3482	297.0781636	1.4619	0.00013607	0.016516
**ENSAPLG00000012449**	148.6337193	691.6009511	-2.2182	1.83E-05	0.0038945
**ENSAPLG00000012560**	49.43557556	0.866923441	5.8335	3.30E-05	0.0061111
**ENSAPLG00000012662**	123.8907763	396.2938284	-1.6775	6.09E-05	0.0094609
**ENSAPLG00000012741**	334.8132336	1205.760056	-1.8485	8.01E-05	0.01122
**ENSAPLG00000012804**	77.73262655	13.89459796	2.484	0.00013202	0.016138
**ENSAPLG00000012934**	129.4646671	35.09848788	1.8831	0.00030345	0.028425
**ENSAPLG00000012949**	547.9944018	1421.552254	-1.3752	0.00050176	0.04138
**ENSAPLG00000013017**	1292.82058	533.6715683	1.2765	0.00053141	0.042404
**ENSAPLG00000013058**	69.16805404	265.3241823	-1.9396	0.00027655	0.026481
**ENSAPLG00000013183**	44.90301805	169.0573331	-1.9126	0.00012694	0.015689
**ENSAPLG00000013513**	414.4197341	27.59861261	3.9084	2.48E-05	0.0048542
**ENSAPLG00000013523**	195.6319775	554.71596	-1.5036	0.00022818	0.024277
**ENSAPLG00000013867**	336.8430616	100.532034	1.7444	5.38E-05	0.0086973
**ENSAPLG00000013979**	4067.123841	11767.42589	-1.5327	3.62E-05	0.0065952
**ENSAPLG00000014002**	48.24332775	169.0096873	-1.8087	0.00026415	0.026167
**ENSAPLG00000014022**	40.68990233	141.2846979	-1.7959	0.00069062	0.049392
**ENSAPLG00000014065**	189.4619627	61.24186639	1.6293	0.00041862	0.036255
**ENSAPLG00000014123**	1262.184287	291.2200332	2.1157	5.31E-08	3.71E-05
**ENSAPLG00000014135**	258.237629	72.25708125	1.8375	4.00E-05	0.0070338
**ENSAPLG00000014191**	941.5950531	2452.800683	-1.3813	0.00016284	0.018108
**ENSAPLG00000014194**	228.7936011	57.38370748	1.9953	2.25E-05	0.0045523
**ENSAPLG00000014222**	10.71263439	62.44231063	-2.5432	0.00026903	0.026347

**ENSAPLG00000014281**	2696.935297	896.0888695	1.5896	0.00032026	0.029525
**ENSAPLG00000014297**	904.4592587	257.3458124	1.8133	3.73E-06	0.0010608
**ENSAPLG00000014378**	469.2657128	1417.477923	-1.5948	2.38E-05	0.0047174
**ENSAPLG00000014394**	13.12731631	73.92672159	-2.4935	0.0001851	0.020321
**ENSAPLG00000014473**	123.7624973	340.6988758	-1.4609	0.00064401	0.047371
**ENSAPLG00000014516**	71.76383666	379.0951075	-2.4012	6.22E-08	3.83E-05
**ENSAPLG00000014519**	74.13575892	316.2841183	-2.093	3.75E-06	0.0010608
**ENSAPLG00000014674**	30.28664762	114.043429	-1.9128	0.00042958	0.036474
**ENSAPLG00000014730**	5575.849583	2231.355193	1.3213	0.00032033	0.029525
**ENSAPLG00000014871**	21.10582701	104.2068718	-2.3037	7.66E-05	0.010917
**ENSAPLG00000014945**	1341.312914	477.281161	1.4907	7.18E-05	0.01048
**ENSAPLG00000015178**	883.3182399	2484.685654	-1.4921	0.00020761	0.022365
**ENSAPLG00000015201**	12.98897524	231.5638503	-4.1561	7.69E-14	1.89E-10
**ENSAPLG00000015224**	2911.925029	404.7878869	2.8467	3.31E-08	2.48E-05
**ENSAPLG00000015225**	67.32686057	388.3506162	-2.5281	2.91E-08	2.39E-05
**ENSAPLG00000015230**	1614.428511	3983.94786	-1.3032	0.00045188	0.037993
**ENSAPLG00000015275**	3608.658794	1042.897294	1.7909	3.25E-05	0.0060863
**ENSAPLG00000015286**	186.3580867	55.44271656	1.749	0.00027594	0.026481
**ENSAPLG00000015349**	248.3651031	894.9281959	-1.8493	0.00037983	0.033746
**ENSAPLG00000015389**	674.6394259	268.0077562	1.3318	0.00054467	0.042672
**ENSAPLG00000015492**	242.036623	69.47502613	1.8007	8.97E-05	0.012081
**ENSAPLG00000015498**	63.23196171	10.4269042	2.6003	0.00015085	0.017688
**ENSAPLG00000015646**	52.37092416	169.9076958	-1.6979	0.00067085	0.048583
**ENSAPLG00000015665**	13.49957984	97.99343389	-2.8598	6.08E-06	0.0015649
**ENSAPLG00000015677**	107.4357236	366.3015895	-1.7696	5.40E-05	0.0086973
**ENSAPLG00000015749**	168.3460736	651.8747088	-1.9532	3.67E-06	0.0010608
**ENSAPLG00000015868**	1225.169221	319.7901594	1.9378	2.86E-05	0.0054143
**ENSAPLG00000015891**	332.3432016	1267.741586	-1.9315	0.0003411	0.030943
**ENSAPLG00000015932**	1404.248053	512.6283298	1.4538	0.00014224	0.017025
**ENSAPLG00000016034**	378.2649176	1530.247403	-2.0163	2.20E-07	0.00010854
**ENSAPLG00000016039**	391.1482493	106.4244392	1.8779	7.25E-06	0.0017601
**ENSAPLG00000016231**	1199.721999	397.0012536	1.5955	7.19E-05	0.01048
**ENSAPLG00000016282**	17.90385838	131.1291443	-2.8726	4.83E-07	0.00020312
**ENSAPLG00000016328**	235.7056317	708.6648797	-1.5881	7.24E-05	0.01048
**Novel00036**	56.2645981	9.55998076	2.5571	0.00042753	0.036474
**Novel00055**	23.47774926	0		0.00016784	0.018544
**Novel00088**	85.9853081	823.3091075	-3.2593	3.21E-08	2.48E-05
**Novel00093**	4.223177821	54.5208851	-3.6904	1.96E-05	0.0041105
**Novel00149**	690.2115934	150.4542135	2.1977	6.99E-05	0.01044
**Novel00165**	6.03167373	147.7840108	-4.6148	3.73E-11	6.43E-08
**Novel00194**	61.82842689	8.247684142	2.9062	6.44E-05	0.0099086
**Novel00263**	301.4730035	100.5631191	1.5839	0.00032353	0.029549
**Novel00264**	539.9026967	208.9616706	1.3695	0.00070105	0.049865
**Novel00337**	80.90440889	286.4897249	-1.8242	0.00010083	0.013353
**Novel00340**	344.5650143	920.1901266	-1.4172	0.00026381	0.026167
**Novel00446**	121.730141	30.02332401	2.0195	0.00031327	0.029186
**Novel00554**	75.65751061	15.11160292	2.3238	0.00025885	0.026021
**Novel00571**	346.3735102	127.8520338	1.4379	0.00059026	0.044427
**Novel00631**	204.7499142	33.0311202	2.632	1.18E-07	6.36E-05
**Novel00701**	773.4074448	136.36177	2.5038	9.40E-10	1.28E-06
**Novel00793**	1468.657195	227.1133081	2.693	4.38E-12	8.39E-09
**Novel00890**	2.553022974	33.34081809	-3.707	0.00061714	0.045849
**Novel00913**	44.21131274	175.1993022	-1.9865	7.34E-05	0.010544
**Novel00948**	139.7622784	41.61232515	1.7479	0.00055068	0.042755
**Novel00959**	24.63729952	0.445373178	5.7897	0.00024642	0.025472
**Novel01018**	595.636566	123.9555276	2.2646	5.88E-08	3.76E-05
**Novel01095**	1287.82016	409.0821205	1.6545	1.69E-05	0.0036393
**Novel01269**	50.46684684	236.2744082	-2.2271	2.26E-06	0.00076374
**Novel01374**	601.3311851	1434.212403	-1.254	0.00051234	0.041459
**Novel01381**	12.5211303	123.8654618	-3.3063	1.53E-05	0.0034154
**Novel01387**	726.0469852	289.8104086	1.325	0.00063272	0.046805
**Novel01402**	342.44965	108.2939613	1.6609	0.00010875	0.014093
**Novel01417**	5.287146666	171.3556677	-5.0184	9.43E-15	3.12E-11
**Novel01448**	375.4150883	67.62932688	2.4728	2.03E-08	1.75E-05
**Novel01458**	165.0686477	452.6531505	-1.4553	0.00055009	0.042755
**Novel01459**	230.7831977	72.51187112	1.6702	0.00068166	0.04916
**Novel01463**	38.80846047	218.9888111	-2.4964	1.29E-07	6.60E-05
**Novel01543**	887.8885005	224.313539	1.9849	0.00015632	0.017751
**Novel01739**	571.0747408	13.07532035	5.4488	1.69E-08	1.54E-05
**Novel01796**	88.72194307	262.0273216	-1.5624	0.0002951	0.027794
**Novel01929**	117.4767769	597.4418532	-2.3464	1.30E-07	6.60E-05
**Novel02015**	17990.64086	3164.256034	2.5073	5.13E-05	0.0084134
**Novel02018**	8.169673517	58.80785647	-2.8477	0.00029465	0.027794
**Novel02155**	154.2101214	38.82097151	1.99	0.00024439	0.025472

Differential expression genes between Native Pekin and Cherry Valley Pekin ducks in 3-week old(BD3, CD3).

**Gene_id**	readcount_BD3	readcount_CD3	log2FoldChange	pval	padj
**ENSAPLG00000001996**	44.41497221	246.4812096	-2.4724	0.00014019	0.047484
**ENSAPLG00000002226**	1.980737429	47.16407054	-4.5736	0.00011486	0.043133
**ENSAPLG00000002581**	4530.58408	1016.51727	2.1561	3.34E-05	0.019259
**ENSAPLG00000002687**	45148.94432	1805.542776	4.6442	2.16E-17	3.72E-13
**ENSAPLG00000002688**	24.12189414	157.6211985	-2.708	5.39E-05	0.025864
**ENSAPLG00000003004**	6.113861038	167.2912613	-4.7741	3.46E-09	9.96E-06
**ENSAPLG00000003383**	380.6811354	1569.408361	-2.0436	0.00010145	0.039978
**ENSAPLG00000004243**	114420.1747	24108.6091	2.2467	4.23E-06	0.004322
**ENSAPLG00000004541**	6469.243058	1311.342133	2.3026	4.51E-06	0.0043312
**ENSAPLG00000004963**	2853.277633	672.3137738	2.0854	4.37E-05	0.022199
**ENSAPLG00000005008**	72.4544775	697.8956072	-3.2679	8.17E-08	0.00017307
**ENSAPLG00000005481**	13423.00054	3481.78446	1.9468	7.78E-05	0.033615
**ENSAPLG00000005651**	618.7752154	4713.215245	-2.9292	1.84E-05	0.012479
**ENSAPLG00000005689**	313.8158252	49.00040381	2.6791	3.53E-06	0.0043003
**ENSAPLG00000005735**	73.27266363	5.196723089	3.8176	3.60E-05	0.019503
**ENSAPLG00000006068**	34.3591313	0		0.00010183	0.039978

**ENSAPLG00000006683**	11.34023585	116.7816807	-3.3643	1.20E-05	0.0097929
**ENSAPLG00000008327**	49.84711199	0.39223065	6.9897	7.14E-07	0.0010846
**ENSAPLG00000008390**	2484.108847	563.5204022	2.1402	1.76E-05	0.012479
**ENSAPLG00000008655**	22.66097671	302.8620343	-3.7404	1.47E-08	3.63E-05
**ENSAPLG00000008717**	4969.708319	880.5991325	2.4966	0.00012793	0.046039
**ENSAPLG00000009922**	38754.51677	5963.178823	2.7002	5.36E-07	0.00092656
**ENSAPLG00000010806**	16055.84201	4089.167382	1.9732	4.11E-05	0.021533
**ENSAPLG00000011054**	17.64036687	144.8132905	-3.0372	2.89E-05	0.017238
**ENSAPLG00000011553**	12742.49773	2777.13409	2.198	1.25E-05	0.0097929
**ENSAPLG00000012106**	185.7241772	956.3920442	-2.3644	0.00010469	0.040188
**ENSAPLG00000012198**	24284.12867	4338.32763	2.4848	7.53E-07	0.0010846
**ENSAPLG00000012263**	95.51236292	609.9313298	-2.6749	0.00013954	0.047484
**ENSAPLG00000012398**	346.6398652	22.72361445	3.9312	3.37E-10	1.46E-06
**ENSAPLG00000012450**	18344.49264	4882.058537	1.9098	8.69E-05	0.036595
**ENSAPLG00000012741**	195.3479255	1107.23028	-2.5028	1.02E-06	0.0013527
**ENSAPLG00000012773**	2.87797115	99.42713954	-5.1105	0.00012343	0.045364
**ENSAPLG00000013360**	8668.595592	2446.85165	1.8249	0.00014867	0.049387
**ENSAPLG00000014123**	1788.587936	266.9451685	2.7442	9.02E-08	0.00017307
**ENSAPLG00000014322**	278.5935764	54.1264976	2.3638	6.15E-05	0.027976
**ENSAPLG00000014519**	53.62231686	290.7137677	-2.4387	5.19E-05	0.025608
**ENSAPLG00000015082**	1957.781289	109.9331901	4.1545	2.52E-13	2.17E-09
**ENSAPLG00000015201**	25.00450663	212.7615892	-3.089	4.25E-06	0.004322
**Novel00008**	74.48882613	0.39223065	7.5692	2.03E-09	7.01E-06
**Novel00088**	136.9800639	755.1208659	-2.4627	3.73E-06	0.0043003
**Novel00093**	1.260026205	50.14178111	-5.3145	2.59E-05	0.01599
**Novel00446**	180.2969111	27.36797225	2.7198	1.88E-05	0.012479
**Novel00881**	23.40605666	371.6327732	-3.9889	1.68E-10	9.67E-07
**Novel00913**	24.10239916	161.3222925	-2.7427	9.32E-05	0.038313
**Novel00980**	0	47.94853184		6.13E-06	0.0055686
**Novel01138**	0	42.94725803		6.90E-06	0.0059579
**Novel01417**	22.30793172	157.2385193	-2.8173	6.07E-05	0.027976
**Novel01703**	856.1312245	210.1856607	2.0262	0.00013595	0.047484
**Novel01739**	103.6459513	11.98679994	3.1121	3.61E-05	0.019503
**Novel01795**	121.6539844	14.02124667	3.1171	1.78E-05	0.012479
**Novel02015**	12510.58301	2908.215312	2.1049	2.48E-05	0.015858
**Novel02155**	203.197769	35.75273496	2.5068	6.68E-05	0.029592

Differential expression genes between Native Pekin and Charry Valley Pekin ducks in 6-week old.(BD6, CD6)

**Gene_id**	readcount_BD6	readcount_CD6	log2FoldChange	pval	padj
**ENSAPLG00000000583**	3626.675847	917.2953488	1.9832	1.04E-07	4.76E-05
**ENSAPLG00000000788**	101.8748292	14.23024742	2.8398	1.96E-06	0.00055581
**ENSAPLG00000000798**	364.5203782	102.1678598	1.8351	1.19E-05	0.0024369
**ENSAPLG00000000930**	47.32478604	218.2667222	-2.2054	5.74E-05	0.0087876
**ENSAPLG00000001031**	7928.128026	2130.923571	1.8955	2.38E-05	0.004252
**ENSAPLG00000001082**	1.0013397	137.0717517	-7.0969	2.07E-05	0.0037355
**ENSAPLG00000001519**	0	41.46824632		2.01E-08	1.27E-05
**ENSAPLG00000001671**	143.3158804	23.71830082	2.5951	1.01E-06	0.00032946
**ENSAPLG00000001855**	1007.12617	164.1717822	2.617	0.00030128	0.029087
**ENSAPLG00000001869**	264.5368324	75.77862974	1.8036	5.55E-05	0.0086595
**ENSAPLG00000001996**	57.23339378	175.809248	-1.6191	0.00050771	0.042497
**ENSAPLG00000002188**	110.2650637	20.19860273	2.4486	0.00010847	0.014746
**ENSAPLG00000002228**	47.31817934	155.7547696	-1.7188	0.00042166	0.037512
**ENSAPLG00000002229**	48.45042912	185.7784991	-1.939	5.64E-05	0.0087196
**ENSAPLG00000002245**	303.7067774	101.8197903	1.5767	0.00022514	0.024273
**ENSAPLG00000002300**	60.39444378	4.745859386	3.6697	0.00024848	0.025283
**ENSAPLG00000002357**	266.5338231	1633.67073	-2.6157	2.14E-11	3.03E-08
**ENSAPLG00000002458**	326.8552872	24.05903954	3.764	6.56E-09	4.84E-06
**ENSAPLG00000002538**	2364.41855	735.1916682	1.6853	4.65E-06	0.0010805
**ENSAPLG00000002897**	18.63272406	141.3205771	-2.9231	1.49E-07	6.49E-05
**ENSAPLG00000002906**	1461.444923	4473.264364	-1.6139	1.14E-05	0.0023721
**ENSAPLG00000002961**	7.264591354	93.43210073	-3.685	3.69E-08	2.02E-05
**ENSAPLG00000002963**	1429.107199	329.2679904	2.1178	2.07E-07	8.59E-05
**ENSAPLG00000003098**	164.2521323	1008.796644	-2.6187	0.0004637	0.039996
**ENSAPLG00000003132**	77.65291801	11.69244642	2.7315	2.40E-05	0.004252
**ENSAPLG00000003211**	4180.657783	1596.859481	1.3885	0.00032662	0.030932
**ENSAPLG00000003276**	818.4734396	268.4548912	1.6083	0.00029054	0.028537
**ENSAPLG00000003321**	83.30095349	561.8359176	-2.7537	0.00043886	0.038638
**ENSAPLG00000003585**	3233.999955	1285.29233	1.3312	0.00026446	0.02659
**ENSAPLG00000003622**	741.6330208	219.0483395	1.7595	1.37E-05	0.0027296
**ENSAPLG00000003627**	3720.475116	280.3231347	3.7303	2.62E-19	4.45E-15
**ENSAPLG00000003983**	1600.266367	332.1905146	2.2682	8.36E-05	0.012032
**ENSAPLG00000004059**	2145.394195	820.1712776	1.3872	0.00022691	0.024273
**ENSAPLG00000004090**	6811.762111	1029.477478	2.7261	3.35E-12	5.69E-09
**ENSAPLG00000004158**	10.72018902	85.26668314	-2.9917	3.44E-06	0.00082427
**ENSAPLG00000004485**	532.3665429	34.75126202	3.9373	0.00050671	0.042497
**ENSAPLG00000004523**	939.0275457	290.4291793	1.693	1.74E-05	0.0032787
**ENSAPLG00000004555**	773.3675181	274.0495194	1.4967	9.80E-05	0.01343
**ENSAPLG00000004562**	1886.648604	613.4455467	1.6208	1.94E-05	0.0035419
**ENSAPLG00000004563**	7716.834028	1848.003708	2.062	6.90E-06	0.0015148
**ENSAPLG00000004584**	16.66277218	121.7741288	-2.8695	4.90E-07	0.00017726
**ENSAPLG00000004634**	741.8227792	217.0063977	1.7733	6.95E-06	0.0015148
**ENSAPLG00000004669**	144.4220094	23.27375684	2.6335	9.47E-07	0.00031549
**ENSAPLG00000004686**	181.7267917	57.70260699	1.6551	0.00034599	0.032126
**ENSAPLG00000004689**	60.66287063	208.7335095	-1.7828	0.00013424	0.01728
**ENSAPLG00000004787**	316.4689801	105.80969	1.5806	0.00015091	0.018755
**ENSAPLG00000004808**	2737.505402	1088.528519	1.3305	0.00024763	0.025283
**ENSAPLG00000004813**	888.7056532	258.3525119	1.7824	6.14E-06	0.001392
**ENSAPLG00000004868**	1117.804396	3017.180024	-1.4325	9.24E-05	0.012866
**ENSAPLG00000004959**	545.7493445	176.7313243	1.6267	4.68E-05	0.0076415
**ENSAPLG00000005038**	378.8006482	135.7967657	1.48	0.00034184	0.031915
**ENSAPLG00000005104**	2025.915326	229.4694653	3.1422	0.00039629	0.035691
**ENSAPLG00000005150**	0.968612181	29.34225204	-4.9209	5.30E-05	0.0085559
**ENSAPLG00000005402**	0	56.13937234		1.27E-10	1.43E-07

**ENSAPLG00000005477**	39.17655167	146.0297828	-1.8982	0.00013303	0.01728
**ENSAPLG00000005501**	128.2696641	409.7619817	-1.6756	4.55E-05	0.0075143
**ENSAPLG00000005523**	2547.244253	585.5260704	2.1211	3.22E-08	1.82E-05
**ENSAPLG00000005631**	12.95196106	69.50252404	-2.4239	0.00021458	0.023676
**ENSAPLG00000005651**	38.82976235	392.0608609	-3.3358	9.76E-05	0.01343
**ENSAPLG00000005735**	230.5372236	51.44482814	2.1639	2.61E-05	0.0045714
**ENSAPLG00000005933**	252.0757842	40.26774262	2.6462	2.04E-08	1.27E-05
**ENSAPLG00000006093**	910.7612474	349.028205	1.3837	0.00035609	0.032884
**ENSAPLG00000006249**	0	30.3278145		3.40E-06	0.00082427
**ENSAPLG00000006292**	201.9889791	567.0274959	-1.4891	0.00024012	0.025283
**ENSAPLG00000006318**	56.74248099	461.6428482	-3.0243	9.18E-05	0.012866
**ENSAPLG00000006380**	2733.756175	1134.802446	1.2684	0.00053048	0.044186
**ENSAPLG00000006597**	12426.21068	3085.805912	2.0097	5.12E-08	2.63E-05
**ENSAPLG00000006689**	1147.784515	329.351119	1.8012	2.36E-06	0.0006092
**ENSAPLG00000006824**	186.6353077	25.92635921	2.8477	2.10E-08	1.27E-05
**ENSAPLG00000006875**	58.88267717	200.9613207	-1.771	0.00011582	0.015619
**ENSAPLG00000007042**	0.517033609	219.9862522	-8.7329	4.37E-05	0.0072741
**ENSAPLG00000007123**	114.3490909	345.9383894	-1.5971	0.00015561	0.01916
**ENSAPLG00000007153**	4387.472757	1177.546712	1.8976	1.03E-06	0.0003318
**ENSAPLG00000007160**	9979.554295	3460.896236	1.5278	5.39E-05	0.0085559
**ENSAPLG00000007270**	1431.516128	573.084995	1.3207	0.00044717	0.039167
**ENSAPLG00000007428**	413.0359564	87.71900574	2.2353	1.07E-07	4.77E-05
**ENSAPLG00000007476**	231.4205606	30.67221859	2.9155	1.98E-09	1.77E-06
**ENSAPLG00000007797**	20.05952151	130.5025571	-2.7017	9.31E-07	0.00031549
**ENSAPLG00000007896**	3266.880527	845.5969374	1.9499	2.24E-07	8.86E-05
**ENSAPLG00000007924**	2845.609838	1103.029863	1.3673	0.00018117	0.021085
**ENSAPLG00000008117**	2538.299486	895.7324756	1.5027	0.00039699	0.035691
**ENSAPLG00000008219**	1185.022924	453.0035636	1.3873	0.00020754	0.023201
**ENSAPLG00000008253**	315.2973961	82.71055529	1.9306	5.35E-05	0.0085559
**ENSAPLG00000008273**	189.7831443	734.5003695	-1.9524	1.43E-06	0.00044096
**ENSAPLG00000008403**	239.3006741	3081.430114	-3.6867	1.51E-08	1.03E-05
**ENSAPLG00000008411**	483.0450722	150.7231521	1.6803	2.69E-05	0.0046614
**ENSAPLG00000008432**	14743.42495	2523.994477	2.5463	2.24E-07	8.86E-05
**ENSAPLG00000008567**	243.0309994	1930.751058	-2.9899	1.16E-13	3.81E-10
**ENSAPLG00000008595**	72.05712416	236.0459907	-1.7119	0.00013356	0.01728
**ENSAPLG00000008874**	0	57.02846029		1.10E-10	1.33E-07
**ENSAPLG00000008884**	2988.508703	1207.047891	1.3079	0.00043058	0.038106
**ENSAPLG00000009157**	535.3825515	206.4399727	1.3748	0.00050403	0.042497
**ENSAPLG00000009268**	410.9486138	116.8279897	1.8146	1.43E-05	0.0027778
**ENSAPLG00000009305**	148.9573092	37.72261088	1.9814	0.00033558	0.031503
**ENSAPLG00000009460**	1827.805261	360.9319273	2.3403	2.28E-09	1.94E-06
**ENSAPLG00000009522**	28.10296663	437.8475746	-3.9616	1.06E-16	6.00E-13
**ENSAPLG00000009599**	6536.124349	2494.385687	1.3898	0.00019706	0.022323
**ENSAPLG00000009602**	1360.434528	543.3910081	1.324	0.00049067	0.041897
**ENSAPLG00000009749**	421.8453479	138.2380922	1.6096	8.01E-05	0.011637
**ENSAPLG00000009777**	1029.927278	355.8636561	1.5331	7.71E-05	0.011297
**ENSAPLG00000009831**	41.63741634	139.5240743	-1.7446	0.0003928	0.035691
**ENSAPLG00000009943**	68.4378889	12.24812639	2.4822	0.00045315	0.039486
**ENSAPLG00000009955**	38.82315565	132.8290823	-1.7746	0.00057666	0.047602
**ENSAPLG00000009972**	178.4870733	31.30971159	2.5111	1.50E-06	0.00045606
**ENSAPLG00000010071**	124.5000047	516.6463779	-2.053	5.20E-07	0.00018409
**ENSAPLG00000010148**	94.21750763	352.3732809	-1.903	1.21E-05	0.0024533
**ENSAPLG00000010246**	512.9282323	32.9877476	3.9588	1.99E-17	1.69E-13
**ENSAPLG00000010278**	643.7103908	176.6128576	1.8658	3.30E-06	0.00081291
**ENSAPLG00000010280**	118.4592389	16.87918441	2.8111	3.17E-05	0.0054463
**ENSAPLG00000010327**	338.1057232	91.43898359	1.8866	1.41E-05	0.0027778
**ENSAPLG00000010515**	550.6716858	165.3759513	1.7354	1.68E-05	0.0032161
**ENSAPLG00000010847**	8709.491212	3486.775979	1.3207	0.00025599	0.025892
**ENSAPLG00000010852**	9.365453311	285.6858104	-4.9309	0.00021661	0.023746
**ENSAPLG00000010949**	314.6500643	814.2260894	-1.3717	0.00029351	0.028663
**ENSAPLG00000010951**	9075.501395	2474.995816	1.8746	3.56E-07	0.00013137
**ENSAPLG00000010999**	1.0013397	1421.007001	-10.471	1.28E-08	9.07E-06
**ENSAPLG00000011010**	5493.771685	2141.370214	1.3593	0.00017537	0.020694
**ENSAPLG00000011011**	1126.185576	307.4231651	1.8731	2.20E-06	0.00058354
**ENSAPLG00000011108**	83.17665012	247.8752307	-1.5754	0.00037886	0.034798
**ENSAPLG00000011263**	97.71904622	12.13332502	3.0097	2.54E-06	0.00064465
**ENSAPLG00000011319**	3112.831551	398.1220253	2.9669	1.57E-13	3.81E-10
**ENSAPLG00000011374**	153.3881258	43.91323818	1.8045	0.00017687	0.020726
**ENSAPLG00000011440**	2336.225844	6405.968079	-1.4552	5.55E-05	0.0086595
**ENSAPLG00000011742**	165.9410559	31.33170381	2.405	1.92E-06	0.00055361
**ENSAPLG00000011749**	33.52190948	259.74963	-2.9539	8.25E-10	7.79E-07
**ENSAPLG00000011811**	77.77722139	238.0537693	-1.6139	0.00031786	0.030515
**ENSAPLG00000012106**	94.74114794	282.4823684	-1.5761	0.00023742	0.025214
**ENSAPLG00000012246**	39.04564159	143.9365257	-1.8822	0.0001657	0.019968
**ENSAPLG00000012276**	13.82239068	110.9597742	-3.005	3.44E-07	0.00012972
**ENSAPLG00000012339**	109.0280246	2276.673189	-4.3842	0.00027163	0.026991
**ENSAPLG00000012650**	22.92602384	137.4491442	-2.5838	2.05E-06	0.00056724
**ENSAPLG00000012760**	165.5810532	43.91323818	1.9148	6.76E-05	0.010082
**ENSAPLG00000012810**	247.0627849	1263.14826	-2.3541	2.12E-06	0.00057198
**ENSAPLG00000012879**	1070.941954	326.1171791	1.7154	7.36E-06	0.0015796
**ENSAPLG00000012893**	6806.078719	830.8476637	3.0342	4.71E-06	0.0010805
**ENSAPLG00000012949**	163.3489752	469.9950589	-1.5247	0.00018688	0.021596
**ENSAPLG00000012990**	256.1532047	42.92401035	2.5772	2.55E-08	1.49E-05
**ENSAPLG00000013104**	473.7579813	1312.648655	-1.4703	8.80E-05	0.01256
**ENSAPLG00000013272**	1191.130369	347.6615848	1.7766	0.00024605	0.025283
**ENSAPLG00000013340**	87.08382636	19.53178677	2.1566	0.00017053	0.020263
**ENSAPLG00000013386**	2874.154535	521.1858016	2.4633	1.87E-05	0.0034866
**ENSAPLG00000013416**	569.6644126	205.180823	1.4732	0.00024714	0.025283
**ENSAPLG00000013492**	26422.14481	8490.779422	1.6378	6.70E-06	0.001498
**ENSAPLG00000013801**	1507.782194	376.5702889	2.0014	3.08E-07	0.00011904
**ENSAPLG00000013917**	3.488325189	57.15425777	-4.0343	1.93E-05	0.0035419
**ENSAPLG00000013979**	1329.491792	3489.969178	-1.3923	0.00024777	0.025283
**ENSAPLG00000014001**	334.794555	95.53635386	1.8092	0.00014897	0.01875

**ENSAPLG00000014054**	182.9440107	620.396974	-1.7618	0.00015122	0.018755
**ENSAPLG00000014065**	22.28468685	162.5755592	-2.867	4.83E-08	2.57E-05
**ENSAPLG00000014084**	770.18726	205.2174767	1.9081	1.17E-06	0.00036788
**ENSAPLG00000014120**	27.90660152	104.4504004	-1.9041	0.00041981	0.037512
**ENSAPLG00000014240**	23622.7915	9231.864541	1.3555	0.00021874	0.023826
**ENSAPLG00000014244**	1633.591606	315.0072451	2.3746	4.97E-09	4.02E-06
**ENSAPLG00000014302**	19.57521542	119.3328023	-2.6079	6.05E-05	0.0090977
**ENSAPLG00000014372**	117.3792308	31.55031043	1.8955	0.00032088	0.030631
**ENSAPLG00000014394**	125.2983725	11.25156782	3.4772	5.77E-09	4.46E-06
**ENSAPLG00000014474**	185.4703304	28.4641602	2.704	7.35E-08	3.57E-05
**ENSAPLG00000014523**	18.27932805	88.80837345	-2.2805	0.00014347	0.018192
**ENSAPLG00000014692**	3373.662037	1339.714663	1.3324	0.00019511	0.02225
**ENSAPLG00000014730**	1935.600405	4776.474464	-1.3032	0.00049878	0.042376
**ENSAPLG00000014885**	2424.092989	838.8836184	1.5309	3.84E-05	0.0064664
**ENSAPLG00000015119**	10.11157955	62.88201424	-2.6366	0.00012248	0.016387
**ENSAPLG00000015151**	720.8664012	102.0420623	2.8206	1.85E-11	2.85E-08
**ENSAPLG00000015157**	537.9623367	1458.479473	-1.4389	0.00013055	0.017196
**ENSAPLG00000015509**	524.7998792	172.5826389	1.6045	0.00012837	0.017041
**ENSAPLG00000015868**	2900.597328	1048.553866	1.4679	0.00016405	0.019911
**ENSAPLG00000015886**	705.8529124	273.3277229	1.3687	0.0005771	0.047602
**ENSAPLG00000015937**	24.93530994	113.4120968	-2.1853	7.67E-05	0.011297
**ENSAPLG00000016009**	2262.3257	862.8521986	1.3906	0.0002426	0.025283
**ENSAPLG00000016064**	952.4698076	364.5799134	1.3854	0.00029663	0.028802
**ENSAPLG00000016291**	77.35837033	330.111919	-2.0933	2.37E-06	0.0006092
**ENSAPLG00000016349**	3629.478424	1122.148779	1.6935	7.44E-06	0.0015796
**ENSAPLG00000016360**	18931.18406	4753.985096	1.9936	5.88E-08	2.94E-05
**ENSAPLG00000016379**	263.7973129	716.4816771	-1.4415	0.0001881	0.021596
**Novel00055**	0	20.18027588		0.00045904	0.039796
**Novel00081**	126.8623808	368.997205	-1.5403	0.00026863	0.02685
**Novel00129**	5.42554955	64.8604699	-3.5795	1.68E-06	0.00049089
**Novel00199**	33.98009475	167.8844293	-2.3047	3.90E-06	0.00092108
**Novel00250**	0.517033609	30.77602385	-5.8954	8.79E-06	0.0018432
**Novel00292**	5.392822031	70.50274798	-3.7086	3.19E-06	0.00079748
**Novel00382**	593.7220742	157.7588829	1.9121	2.07E-06	0.00056724
**Novel00414**	399.1222958	135.4743539	1.5588	0.00015706	0.019199
**Novel00522**	3.97263128	386.0851748	-6.6027	1.60E-06	0.00047805
**Novel00531**	152.6552129	40.70129048	1.9071	0.00014333	0.018192
**Novel00546**	942.116534	108.6295838	3.1165	2.14E-13	4.56E-10
**Novel00576**	78.41195168	519.6202172	-2.7283	3.98E-10	3.98E-07
**Novel00701**	144.0355799	662.556519	-2.2016	1.02E-07	4.76E-05
**Novel00820**	1.551100828	26.81178178	-4.1115	0.00038246	0.03494
**Novel00832**	5010.634855	260.486088	4.2657	1.48E-13	3.81E-10
**Novel00947**	56.61817762	10.04373337	2.495	0.00022713	0.024273
**Novel00950**	24.5491864	0.440878604	5.7991	9.23E-05	0.012866
**Novel01047**	0.517033609	20.29507724	-5.2947	0.00046809	0.04017
**Novel01101**	43.61397491	0		0.0001703	0.020263
**Novel01118**	7.748897444	74.88587642	-3.2726	6.04E-05	0.0090977
**Novel01233**	18.24660053	83.82191522	-2.1997	0.00021025	0.02335
**Novel01300**	38.65951806	0		1.59E-07	6.74E-05
**Novel01340**	87.31952569	415.2528047	-2.2496	1.44E-05	0.0027778
**Novel01387**	231.0869847	621.9626992	-1.4284	0.00032767	0.030932
**Novel01448**	80.04802167	321.8720191	-2.0075	3.63E-05	0.0061756
**Novel01487**	268.2674637	54.82406727	2.2908	6.19E-07	0.00021458
**Novel01502**	553.7934016	45.00627126	3.6211	1.54E-15	6.56E-12
**Novel01739**	61.13396333	490.4391007	-3.004	0.0002007	0.022585
**Novel01899**	390.2804828	38.27462548	3.3501	8.11E-13	1.53E-09
**Novel01928**	173.3755856	50.089204	1.7913	0.00029	0.028537
**Novel01929**	1174.400306	100.511816	3.5465	3.99E-11	5.22E-08
**Novel02110**	175.3065092	15.99376183	3.4543	2.94E-10	3.12E-07

Table S8. Primers sequences used for the qRT-PCR validation

Gene	Primers sequence(5'-3')
ACSL1	F:GGGAGGAAGAGTAAGGCTGATG
	R:GGTGCTCCAACATGACCTGC
CAV3	F:ATCAAGGACCAACACACCAAGG
	R:GTCAAAGCTGTACGTTCCCACTG
MYOG	F:GCGGCTGAAGAAGGTGAACGA
	R:ATCTCTCTGCTCCCTCTCCTGCTG
TNNI1	F:CGTGGACAAGCAGTCAGAGAA
	R: ATCTCCCTGGTGTTGTGGTTG
MSTN	F: GCACTGGTATTTGGCAGAGTATT
	R: TCACCTGGTCCTGGGAAAGT
IGFBP5	F: GTCGGAGATGACGGAGGAGA
	R: CGCCATGCCCACAAACTTG
FHL3	F: CGACCTATGAGCCTCTGACGA
	R: GAACCTCCTGAACACACCGC
CD36	F: AGCAGGTCTCCCTCCACACAG
	R: AGCACAGCACCGATGACAGC
GADPH	F:CACAGCCACACACGAAGACA
	R: CCTTAGCCAGCCCCAGTAGA

Figure S1. PCA analysis of two Populations

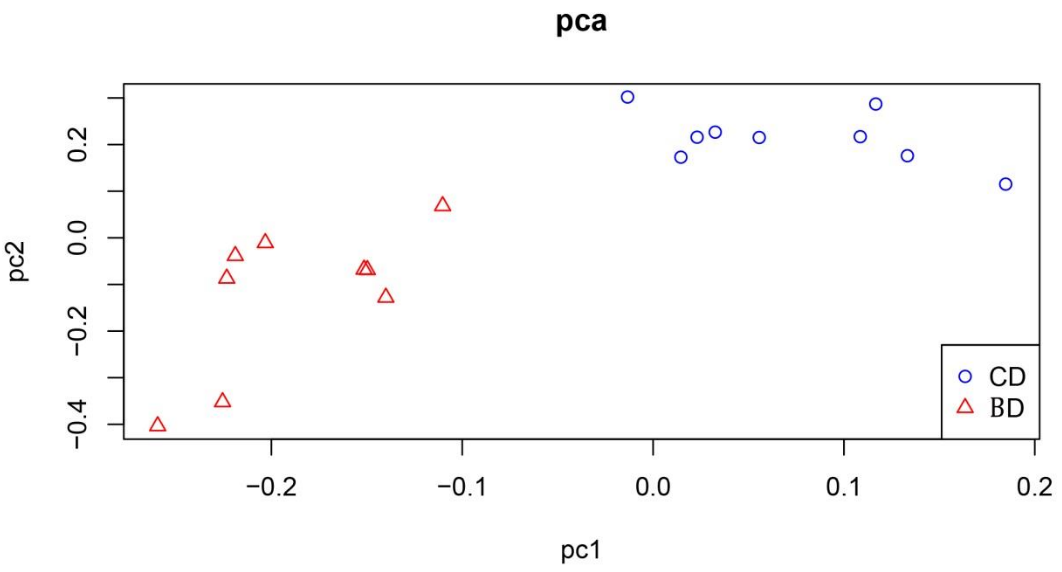

CD: Cherry Valley Pekin ducks; BD: Native Pekin ducks

Figure S2. Visualisation of Main PSGs and Pathways related to Signaling Transduction and Immune

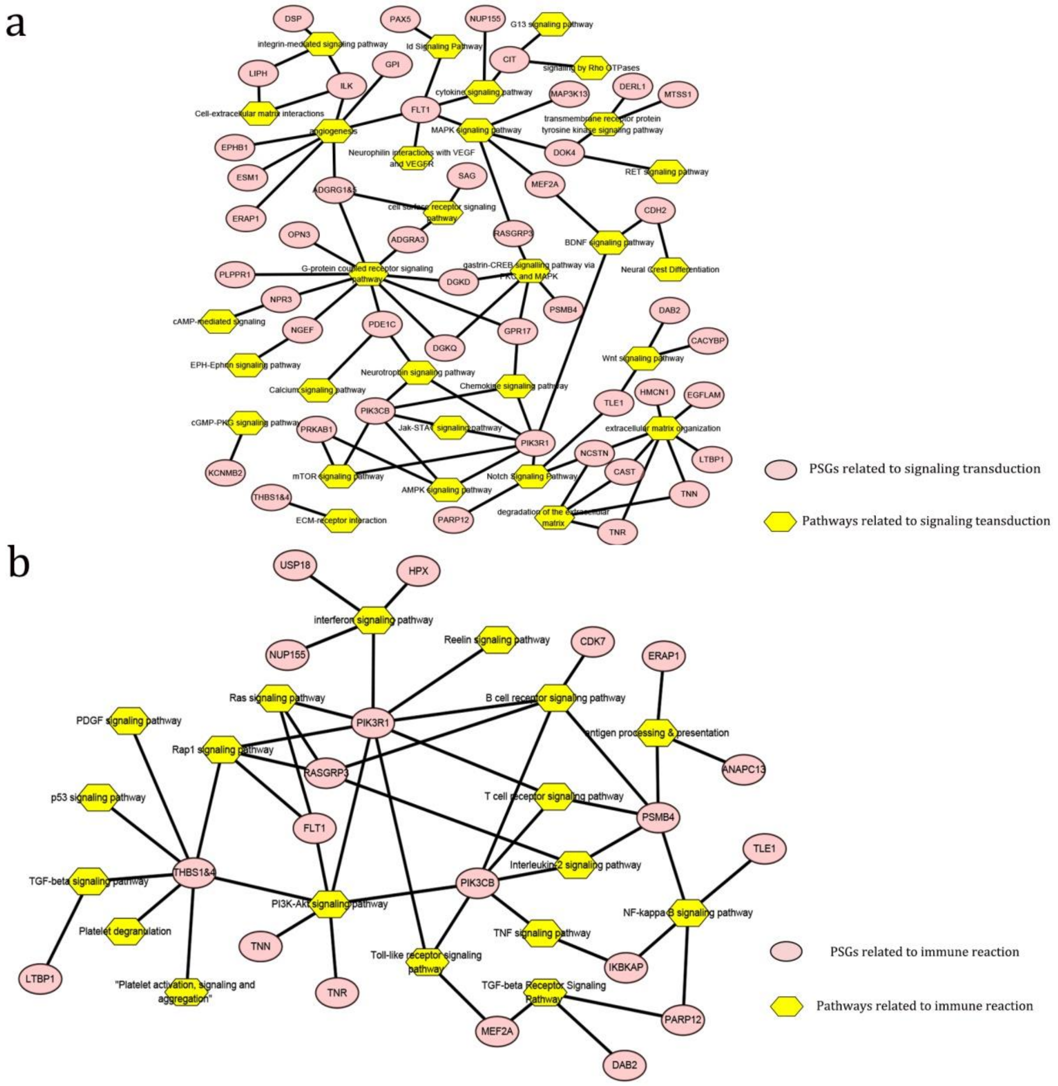

a, main PSGs and pathways related to signaling transduction; b, main PSGs and pathways related to immune system; items in ellipse were positively selected genes while items in rhombus were related pathways.

Figure S3. Overview of positively expressed genes in BD3, BD6, CD3 and CD6

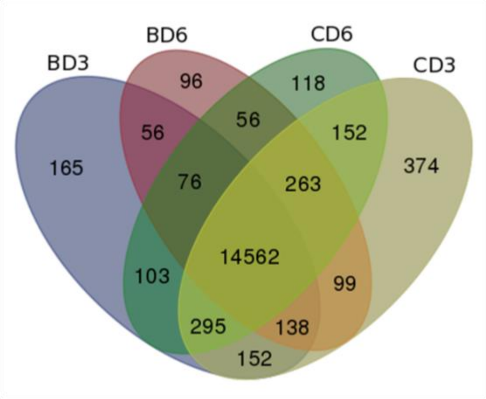

BD3: 3-week age of Native Pekin ducks; BD6: 6-week age of Native Pekin ducks; CD6: 6-week age of Cherry Valley Pekin ducks; CD3: 3-week age of Cherry Valley Pekin ducks

Figure S4. The number of genes detected with different level of RPKM value

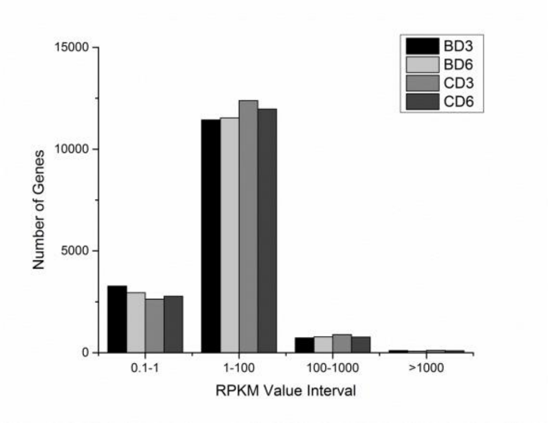

BD3: 3-week age of Native Pekin ducks; BD6: 6-week age of Native Pekin ducks; CD6: 6-week age of Cherry Valley Pekin ducks; CD3: 3-week age of Cherry Valley Pekin ducks

Figure S5. QRT-PCR and sequencing results for eight DEGs

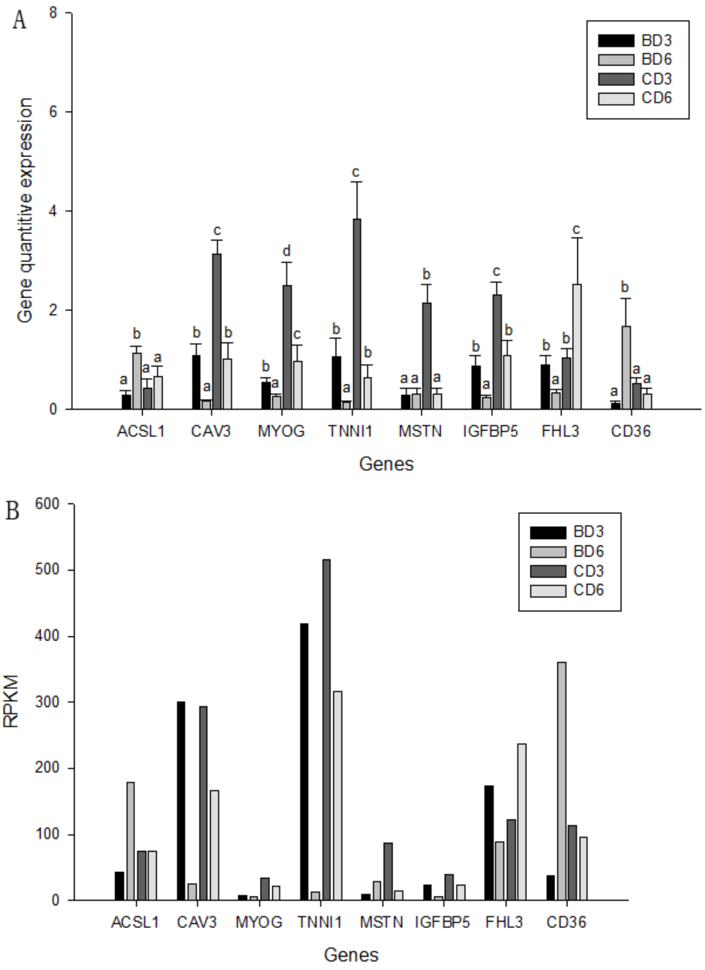

A: Genes quantitative expression of ACSL1, CAV3, MYOG, TNNI1, MSTN, IGFBP5, FHL3, and CD36 using qRT-PCR. Different letter within a gene means significant different expression with $p < 0.05$. B: RPKM values of the eight genes. BD3: 3-week age of Native Pekin ducks; BD6: 6-week age of Native Pekin ducks; CD6: 6-week age of Cherry Valley Pekin ducks; CD3: 3-week age of Cherry Valley Pekin ducks. Similar tendency was observed in qRT-PCR and RNA-seq.
